# Lightweight XOR-based visual cryptography using random shares for secure colour image sharing with minimal shares

**DOI:** 10.1038/s41598-025-27142-2

**Published:** 2025-12-01

**Authors:** Faizal Nujumudeen, D. Muhammad Noorul Mubarak, Tarak Hussain

**Affiliations:** 1 Department of Computer Science, University of Kerala, Thiruvananthapuram, 695581 Kerala India; 2 Department of Computer Science and Engineering, KL Education Foundation, Guntur, 522502 Andhra Pradesh India

**Keywords:** Computational efficiency, Image security, Lightweight cryptography, Lossless reconstruction, Real-time encryption, Visual cryptography, Visual secret sharing, XOR-based Encryption, Engineering, Mathematics and computing

## Abstract

In the current digital environment, safeguarding visual data from unauthorized use remains a substantial challenge. Sensitive imagery, including biometric, medical and defence images, represents a frequent target of destructive cyberattacks. Although conventional visual cryptography approaches achieve satisfactory results in rendering visual content unreadable, these schemes often present serious drawbacks that include excessive computation, pixel expansion and reduced reconstruction quality. This paper suggests a new method and concept of lightweight visual cryptography based on the use of bitwise operations (specifically XOR) for secured colour image sharing. This methodology employs three non-expansible shares while providing the user with lossless encryption and low computation in addition to strong statistical and differential attack resistance during encryption and decryption. The proposed method utilizes a cryptographically secure pseudo-random number generator (CSPRNG) and reversible XOR bitwise operations to determine and generate three unique unreadable shares. Tests and experiments reveal that the resulting shares have high reconstruction quality (PSNR > 40 dB), an ideal degree of randomness (entropy ≈ 7.997) and tolerable processing time (in comparison to traditional XOR-, polynomial- and CRT- based visual cryptography schemes). The new method and concept emphasize the merging of simplicity, scalability, and robustness in a computationally sound and secure solution for real-time use in areas such as military imaging, biometric authentication, and multi-media communication.

## Introduction

In today’s digital world, safeguarding sensitive data has become a critical issue for individuals, businesses, and governments. While conventional security measures such as encryption and password protection provide some level of defence, they are not entirely secure. Advanced threats such as hacking, phishing, and social engineering can often bypass these defences. One promising approach to enhance data security, especially for image-based content, is visual cryptography.

Visual cryptography enables the secure sharing of visual data by breaking an image into several shares^3^, with no single share revealing any information about the original image. Only when the required number of shares is combined can the original image be reconstructed. This technique adds an extra layer of security by ensuring that even if an attacker gains access to one or more shares, they cannot extract meaningful information without the remaining shares. It also added the advantage of less complexity by using visual staking for reconstruction of shares rather than complex calculations^3–11^. But later this was modified with calculations using n-shared concept if VC only^1,2,12–14^. The authors here propose a system to reduce the complexity utmost using simple XOR based calculation.

### Security using images

Images are crucial for storing and representing sensitive data across various fields, including medical imaging, defence, finance, and biometric systems. Protecting the confidentiality of image data is especially important in systems that use facial recognition, fingerprint identification, and medical imaging. For example, biometric data such as fingerprints or retinal scans are saved as images for user authentication purposes. If such data are compromised, they could be used to bypass security systems or steal someone’s identity.

Even though conventional encryption systems such as the Advanced Encryption Standard (AES)^20^ and the Rivest-Shamir-Adleman (RSA) algorithm^21^ can handle the encryption of image data, they might not consistently provide the most effective way to secure visual information. Encrypted images frequently necessitate considerable computational resources for both encoding and decoding processes, which can impede transmission speeds, particularly when dealing with large file sizes. Furthermore, encrypted images must undergo a decryption process prior to being viewed, thereby rendering them potentially vulnerable during both transmission and access by authorized personnel.

Traditional cryptographic techniques, despite their efficacy in securing textual data, encounter significant challenges when extended to the domain of images, primarily due to the inherent high redundancy and sensitivity of images to minimal alterations. Recent innovations in image encryption methodologies, exemplified by those utilizing DNA networks and hyperchaotic systems^1^, exploit the intricate and unpredictable characteristics of these systems to bolster security. In a similar vein, the inter-intra-block scrambling and weighted diffusion^2^ approach offers a robust resolution by disrupting the interrelations of image pixels and infusing controlled randomness. Still, these methods heavily rely on available computational power and the necessary decryption keys, which makes them susceptible to brute-force assaults and various security risks.

In order to proficiently tackle the complexities associated with the protection of images, scholars have increasingly resorted to methodologies such as secret sharing frameworks and visual cryptography. Secret sharing is characterized by the segmentation of data into numerous fragments, necessitating a predetermined quantity of these fragments to reconstruct the original dataset. Visual cryptography broadens this principle to encompass visual assets^3^, presenting an innovative methodology for the protection of images and confining access exclusively to authorized individuals.

In contrast to conventional techniques, visual cryptography obviates the necessity for intricate computations and decryption keys. It disaggregates the confidential image into several shares, each of which appears visually nonsensical in isolation. The original image becomes discernible only when a requisite number of these shares are superimposed. This inherent clarity and security render visual cryptography a compelling strategy for the safeguarding of sensitive image information.

### Visual cryptography: the foundation

Visual Cryptography (VC) constitutes a robust methodology within the domain of image security, which was conceptualized by Moni Naor and Adi Shamir^3^ in the year 1994. Fundamentally, VC is predicated upon the creation of share visual patterns or fragments that emerge from the original confidential image. These shares are disseminated among various recipients or storage sites, whether via secured or unsecured communication channels. A salient characteristic of VC is that individual shares convey no significant information pertaining to the secret image, thereby ensuring absolute confidentiality.

The principal innovation of traditional VC lies in its capacity to reconstruct the secret image utilizing solely human visual perception. Through the overlaying or stacking of the shares, the original image becomes discernible to the unaided eye, negating the necessity for intricate mathematical operations or decryption algorithms. This inherent simplicity rendered early VC methodologies remarkably accessible and efficacious for contexts constrained by limited computational resources.

#### Examples of visual cryptography (black and white)

Consider a black-and-white image, where each pixel in the original image:

- If the pixel is white, a random pattern of black and white subpixels is spread across the shares.
- If the pixel is black, the opposite pattern is used.

**Fig. 1:**
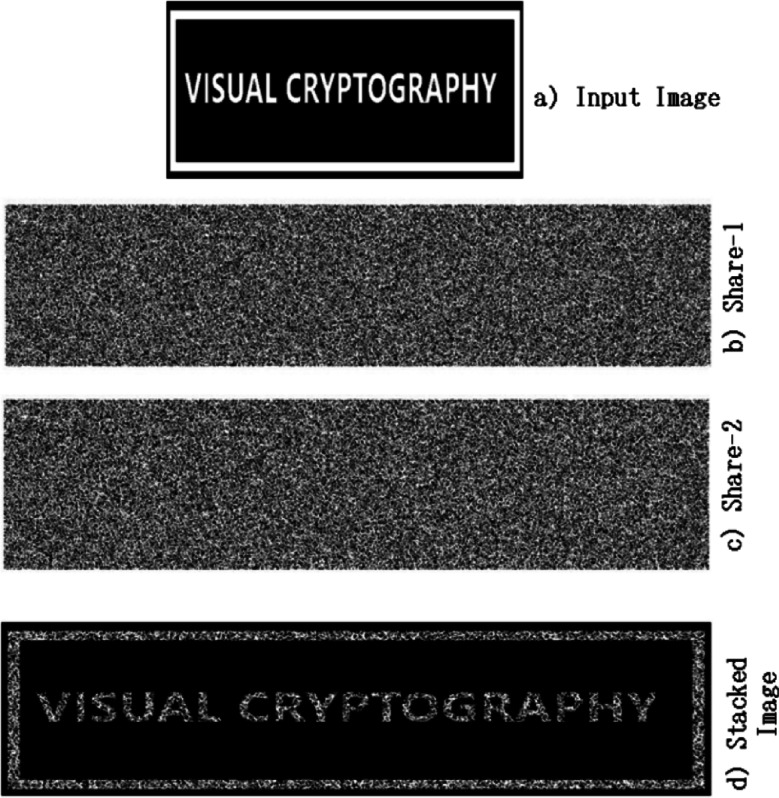
A sample representation of Naor and Shamir’s visual cryptography^3^.

When the shares are combined, the original black and white images reappear. The shares by themselves look like random noise, so you cannot guess what the image looks like just by looking at one share. This makes it very secure. A sample representation of this method is represented in Fig. 1.

### Advancements in visual cryptography

Although the original VC methodologies accentuated visual-based reconstruction, subsequent advancements within the field have introduced computational enhancements to the foundational model^3^. These innovations have broadened the applicability of VC, facilitating colour image processing^4,5,7,9^ adaptable threshold schemes, and superior-quality reconstructions. Computational techniques, particularly those that employ XOR operations^12,14^ have been integrated into VC to surmount limitations such as inadequate resolution, pixel expansion, and the challenges associated with grayscale or colour image processing.

In these advanced VC methodologies, the notion of share generation continues to be pivotal, yet the reconstruction of the secret image frequently depends on mathematical computations^1,2,12–14^ rather than visual perception alone. This transition has permitted enhanced flexibility, improved image quality, and the potential for integration of VC with contemporary cryptographic systems. Nevertheless, this evolution has also departed from the foundational principle of direct visual decryption^3–11^ prioritizing computational efficiency over simplicity.

These advancements have rendered VC increasingly versatile, facilitating its deployment in various domains such as secure multimedia sharing, biometric systems, and medical imaging. However, they have simultaneously introduced novel challenges, including heightened computational demands and the necessity to balance security with reconstruction fidelity.

### Colour visual cryptography

The original method of visual cryptography works with only black and white images^3^. However, as the need to secure coloured images have increased, new methods for colour visual cryptography have been developed. In this technique, each pixel in a colour image is broken down into smaller parts, identical to the black-and-white method, but now across the red, green, and blue (RGB) colour channels^4,5,7,9^. To do this, the system first breaks down each pixel into its basic RGB components. Then, it applies a secret sharing process to each of these components separately. This process creates different shares for each colour channel, which can later be combined to recreate the full-colour image.

In colour visual cryptography, the shares are generated via techniques such as those used for grayscale images. However, instead of just splitting the pixel into black and white, each pixel is split into its red, green, and blue values^5^. The result is that each share appears as a set of random colour values. Only when all the necessary shares are combined do the original colours reappear to form the image. Colour visual cryptography is inherently more complex than its black-and-white counterpart because of the increased amount of information that needs to be stored for each pixel. Managing how colours mix when the shares are overlaid adds an extra layer of challenge to the process, increasing the complexity of colour visual cryptography.

**Fig. 2:**
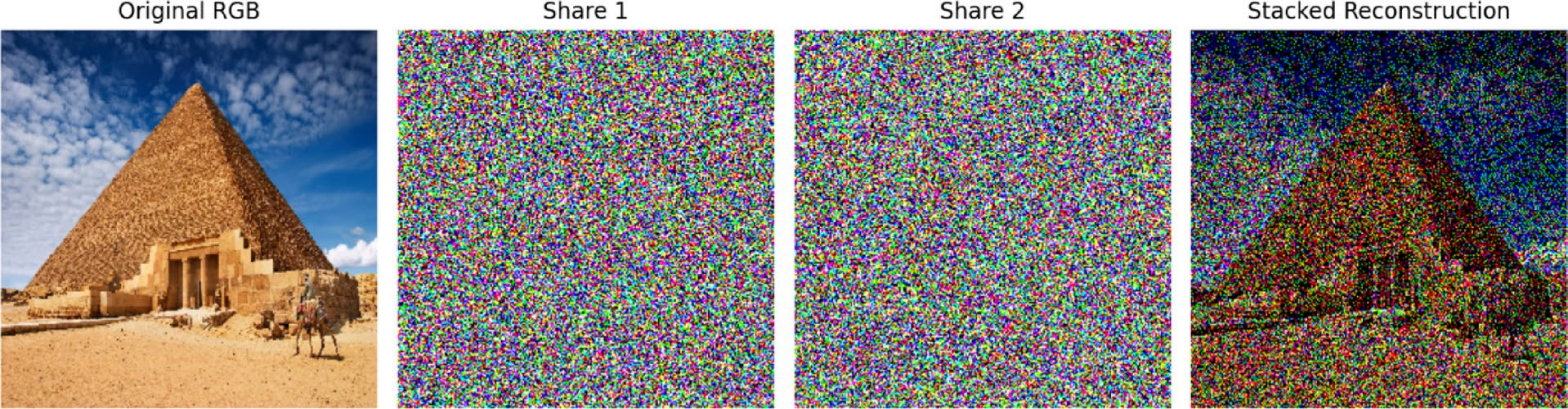
A sample representation of colour visual cryptography^5^.

Figure 1 represents the output of the traditional visual cryptographic scheme formulated by Naor and Shamir^3^, which specifically shows pixel expansion, an extra load in shares, nearly doubled the size of the input image for each share, and the compromised quality of the image. Figure 2 illustrates the colour visual cryptographic scheme devised by Young-Cheng^5^, which clearly indicates the quality of the reconstructed image.

### XOR-based visual cryptography

An advanced method of visual cryptography^12^ uses the XOR (exclusive OR) operation to encode and decode the image shares but using some complex calculations. XOR is a simple but powerful cryptography operation. Here’s how it works:

- (A ⊕ A = 0) (XOR-ing a value with itself yields 0),
- (A ⊕ 0 = A) (XOR-ing a value with 0 yields the value itself),
- XOR is reversible, meaning (A ⊕ B ⊕ B = A).

In XOR-based visual cryptography, each pixel of the image is encrypted by XOR-ing it with random values from different shares. To obtain the original image, XORs are all combined as mentioned in Eq. (1). This method is efficient and keeps the image secure while still making it easy to reconstruct, using the Eq. (2) when all the shares are combined:

1 $${\text{S3}}\left( {{\text{i}},{\text{ j}}} \right) = {\text{P}}\left( {{\text{i}},{\text{ j}}} \right) \oplus {\text{S1}}\left( {{\text{i}},{\text{ j}}} \right) \oplus {\text{S2}}\left( {{\text{i}},{\text{ j}}} \right)$$

To reconstruct the image, we XOR all three shares:

2 $${\text{P}}\left( {{\text{i}},{\text{ j}}} \right) = {\text{S1}}\left( {{\text{i}},{\text{ j}}} \right) \oplus {\text{S2}}\left( {{\text{i}},{\text{ j}}} \right) \oplus {\text{S3}}\left( {{\text{i}},{\text{ j}}} \right)$$

**Fig. 3:**
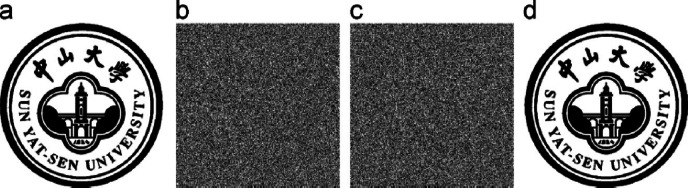
A sample output of XOR-based visual cryptography for grayscale images proposed by Ou et al.^12^. (**a**) Input image, (**b**) share-1, (**c**) share-2, and (**d**) the constructed image.

For example, if a pixel value is XOR-ed with two random values from different shares, the third share can be calculated, and then, by combining them all, the original pixel value is revealed. In this way, the image remains hidden unless all shares are used together. This method enables efficient image encryption and reconstruction while preserving the security features of visual cryptography. Figure 3 shows a representative example of XOR-based visual cryptography applied to a grayscale image, as presented by Ou et al.^12^.

### Motivation of the research

The motivation behind the proposed research stems from the growing need for secure, efficient, and high-quality image protection in an era where sensitive visual data, such as medical scans, biometric images, and confidential media, are frequently transmitted and stored over potentially insecure networks. Traditional visual cryptography methods often suffer from drawbacks like pixel expansion, high computational complexity, and degradation of reconstruction quality, making them less suitable for real-time or resource-constrained environments. The proposed XOR-based colour visual cryptography scheme was conceived to address these limitations by providing a lightweight, non-expansible, and lossless approach that ensures perfect reconstruction while maintaining strong security metrics. This balance of efficiency, quality, and robustness makes it a promising solution for applications ranging from secure multimedia sharing to critical sectors like healthcare, defence, and forensics.

### Problem statement

While visual cryptography has many benefits, several challenges remain, especially regarding securing colour images and ensuring good performance. Some of the main issues include the following:

- *Security of Colour Visual Cryptography*: Binary visual cryptography (for black-and-white images)^1,2^ has proven to be secure, but colour visual cryptography^5–11^ adds complexity. Each pixel now holds more information and handling the random generation of shares across the red, green, and blue (RGB) channels requires careful attention to ensure that no single share leaks any information about the original image.
- *Storage and transmission efficiency*: The use of multiple shares increases the amount of data that needs to be stored or transmitted. Since each share can be as large as the original image, this can create significant overhead in both storage and communication^1,2,8^. This is especially problematic for applications that require efficient data transmission, such as cloud services or mobile devices.
- *Reconstruction Quality*: In some visual cryptography systems, especially those for colour images, the reconstructed image can suffer from a loss in quality^5–7^. Ensuring that the reconstructed image closely matches the original image remains a challenge, particularly when random pixel values are used to generate the shares.
- *Threshold schemes and flexibility*: Most visual cryptography systems rely on a threshold scheme^9–11^, such as a (k, n) scheme, where k shares are needed to reconstruct the image from a total of n shares. Ensuring flexibility in the number of shares without compromising security is crucial for practical applications. For example, in a (3, 3) scheme, all three shares are required to recreate the image. However, more schemes, such as (2, 3), allow any two shares to reconstruct the image, making the system more user friendly while maintaining security.

### Inspiration of the proposed approach

The idea of the proposed scheme of visual cryptography was originally inspired by the work of Naor and Shamir^3^, who introduced a method that allows secret data to be transferred via images without requiring complex calculations while ensuring high levels of security and confidentiality when shares are used. Ou, Sun, and Wu^12^ later updated this strategy and created a non-expansive XOR-based visual cryptography scheme with meaningful shares, which motivated us to develop an algorithm that is both less complex and highly secure for producing meaningful colour shares.

**Fig. 4:**
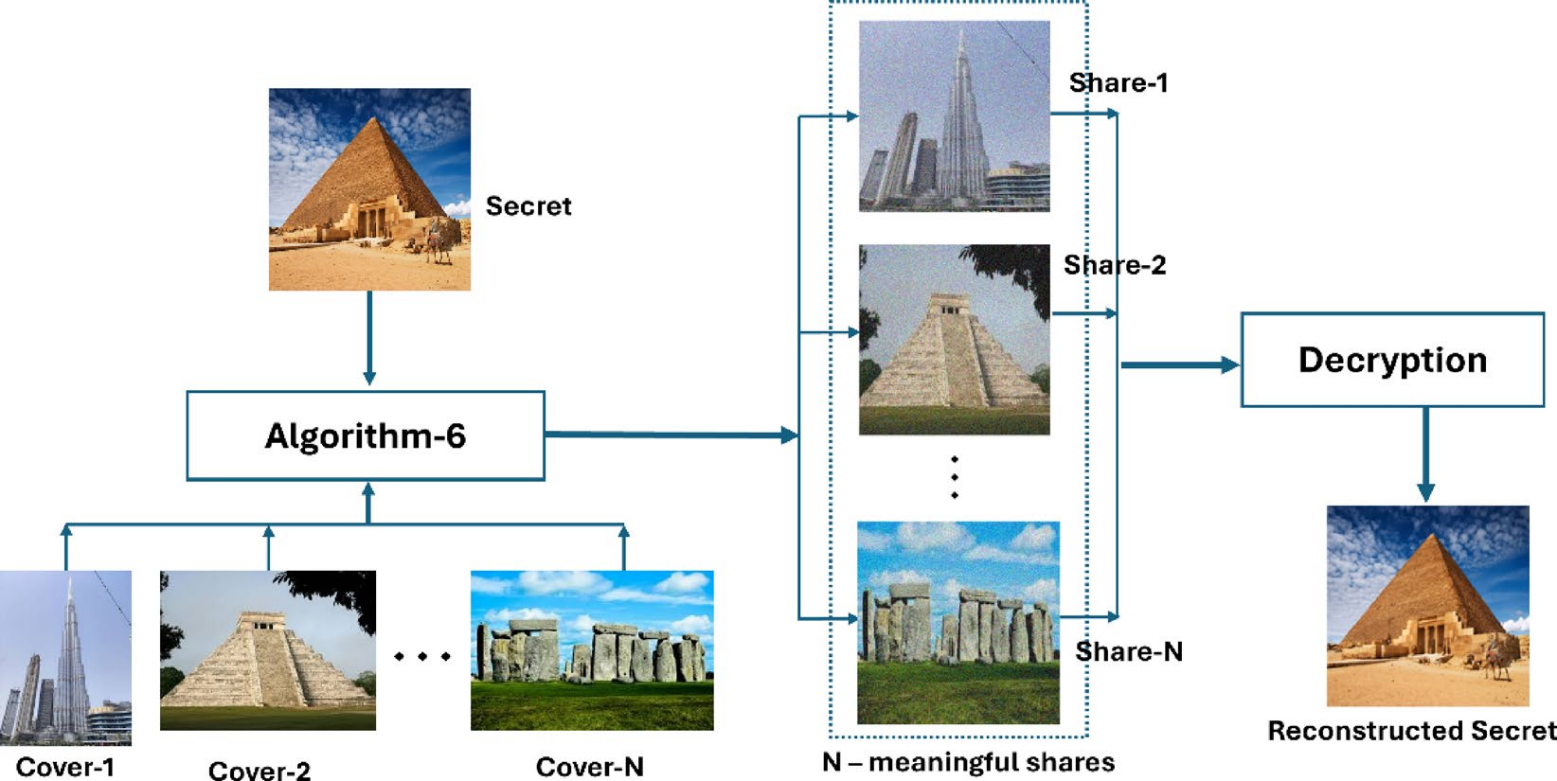
A sample representation of XOR-based (n, n) colour visual cryptography schemes for colour images proposed by Chen et al.^13^ (input image, n-cover images, n-shares and the constructed image).

Moreover, the methodology proposed by Chen and Yuan^13^, titled "XOR-Based (n, n) Visual Cryptography Schemes for Grayscale or Colour Images with Meaningful Shares," influenced our strategy. Figure 4 illustrates Chen and Yuan’s method^13^, which is a variant of XOR-based visual cryptography in which the secret image is distributed among various cover images, forming a collection of significant shares. This approach involves complex calculations and large data transmission overheads.

**Fig. 5:**
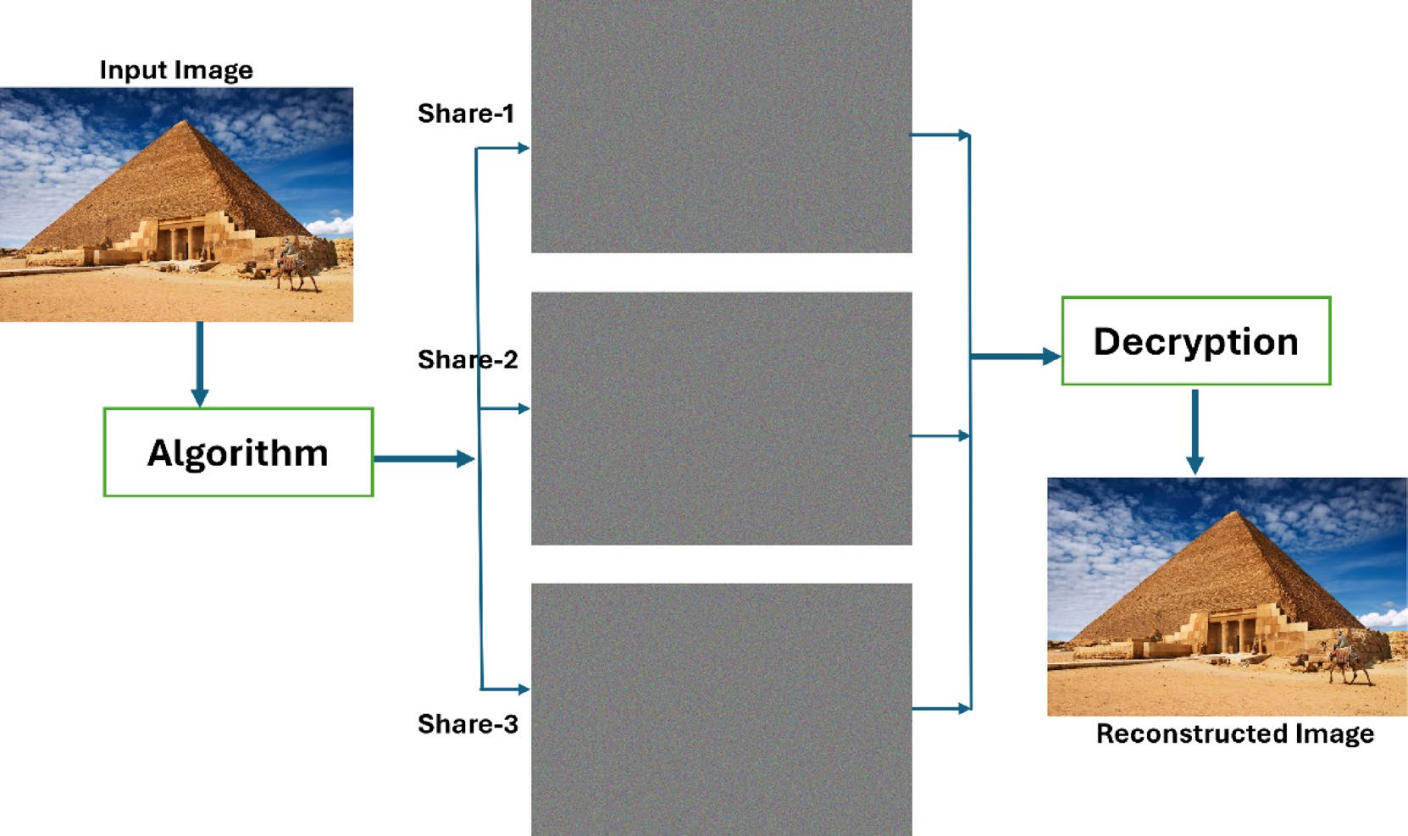
A sample representation of the XOR-based visual cryptography scheme with lossless reconfigurable algorithms proposed by Wang et al.^14^ (input image, 4 shares and the constructed image).

An important source of inspiration arose from the algorithms created by Wang H., Jiang Z., Qian Q., and Wang H.^14^, an efficient XOR-based visual cryptography scheme incorporating lossless reconfigurable algorithms was proposed. We examined the intricacy of these algorithms and their efficacy in maintaining the quality of the reconstructed images. Figure 5 illustrates the sample outcome of Wang et al.'s^14^ methodology, which employed intricate calculations and necessitated a minimum of four shares for representation.

Our goal is to ensure that the generation of shares via XOR-based methods can be simplified while keeping the original image hidden and guaranteeing lossless reconstruction. The authors combined the algorithms developed by Ou, Sun, Wu, Chen and Yuan, and Wang et al.^12^, leading to the development of an algorithm capable of generating three shares from an image without pixel expansion. These shares can be easily combined to reconstruct the original image without any loss of information or quality. The complexity of the algorithm has been reduced, but the qualities of the original techniques, such as no pixel expansion, secure sharing without revealing the secret data or input image, and perfect image restoration, have been preserved.

While visual cryptography has progressed significantly, it still faces important issues. Most of the available schemes utilize complex mathematical operations including polynomial interpolation and modular arithmetic, increasing the computational burden and affecting efficiency in a real time scenario. Full reconstruction errors and pixel expansion are the main limitations of most classical schemes, which results in image quality degradation and storage expansion. For this reason, visual cryptography might not be suitable for practical use in lightweight, lossless and speed-sensitive environments.

This manuscript presents a lightweight XOR-based visual cryptography model that addresses the aforementioned issues by implementing non-expansible share creation and perfect reconstruction with very little computational complexity, while keeping visual cryptography secure. This model allows for efficiency improvements without sacrificing security or fidelity and thereby enhances use cases where bandwidth and resource constraints are paramount. The innovation of this study is incorporating a secure cryptographically pseudo-random number generator with a lightweight optimized framework of XOR encryption that guarantees both security and lightness of computation. The proposed method reduces execution time by over 70% and memory usage by almost 50% while achieving perfect reconstruction quality when compared with existing XOR and polynomial visual cryptography schemes. The proposed system shows strong resistance against known plaintext and ciphertext attacks and, overall, provides a secure, efficient, and scalable platform for visual cryptography applications in real-world scenarios.

### Proposed approach: lightweight XOR-based visual cryptography using random shares for secure colour image sharing with minimal shares

#### Why XOR-based operation?

The XOR operation has key properties that make it useful for secret-sharing schemes:

- *Reversibility*: *A* ⊕ *B* ⊕ *B* = *A*, which means XOR, a value with the same value twice, recovers the original value.
- *No Information in Partial Shares*: No single share or combination of fewer than all the required shares reveal any information about the original image.

These properties guarantee two aspects of the proposed visual cryptography scheme. First, the reassembly of the original image depends solely on the existence of all the generated shares—if there is even one share missing, the original content cannot be recollected or even approximated. Second, when all shares are correctly combined and used, the scheme provides a lossless recollection of the original image, exactly replicating the visual and structural information present in the input with floorless colour fidelity, or as close to the original as defined by the values of the shares. This twofold agreement allows for a versatile and robust methodology in a high security application of visual data.

### Proposed model

**Fig. 6:**
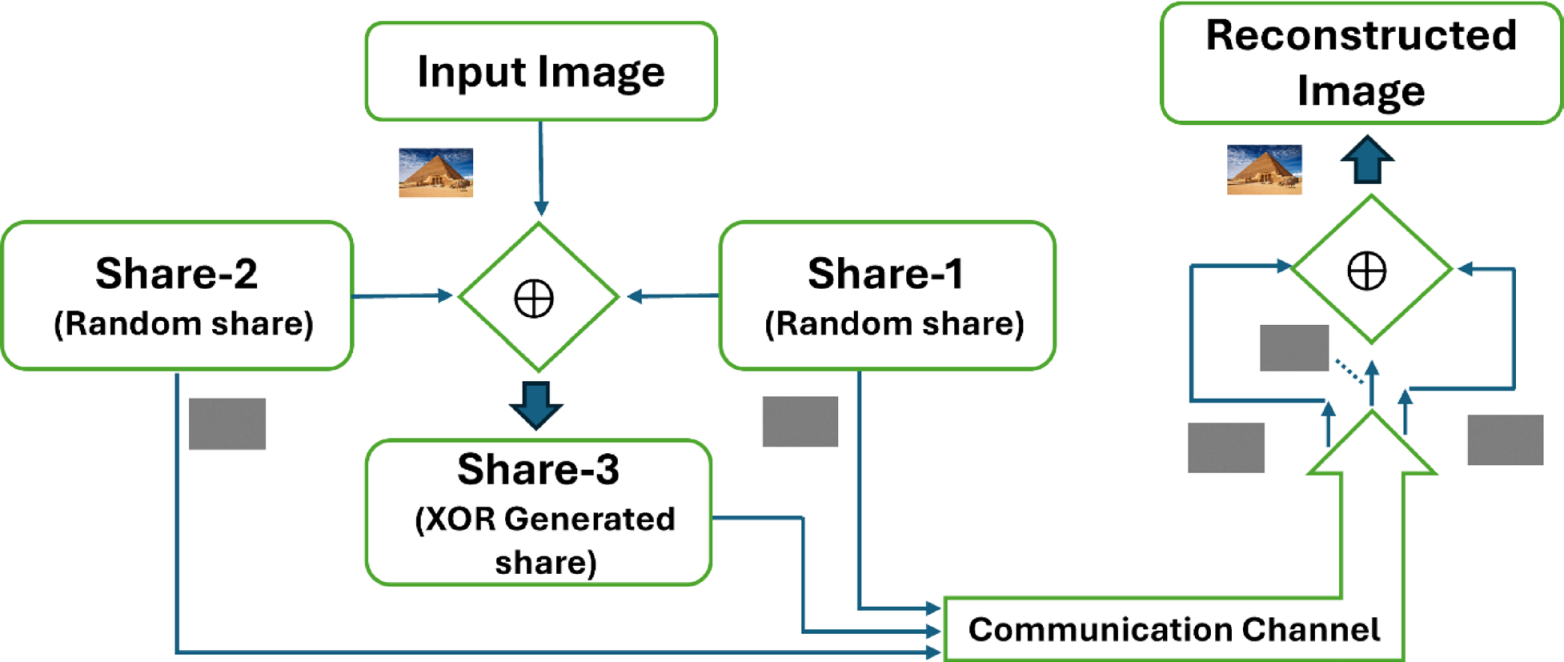
A sample architecture of the proposed system of XOR based VC with random shares.

This paper introduces a new XOR-based (3,3) visual cryptography method specifically for colour images. The basic structure of the architecture is demonstrated using Fig. 6. The approach aims to increase the security and efficiency of visual cryptography and reduce complex calculations while addressing challenges related to image quality and flexibility in the number of shares.

The key contributions of this work include the following:

- *XOR-based share generation*: The RGB values of each pixel are encrypted via XOR operations with randomly generated values for each share. XOR offers a simple, reversible encryption method that ensures that no information is revealed unless all the necessary shares are combined.
- *Secure colour image splitting*: By adapting traditional visual cryptography to work with the RGB colour model, the method ensures that each share contains randomized colour values, making it impossible to deduce the original image from a single share.
- *Efficient reconstruction*: The proposed technique guarantees that the original image is perfectly reconstructed when all shares are combined, ensuring both high security and high-quality image restoration.

**Fig. 7:**
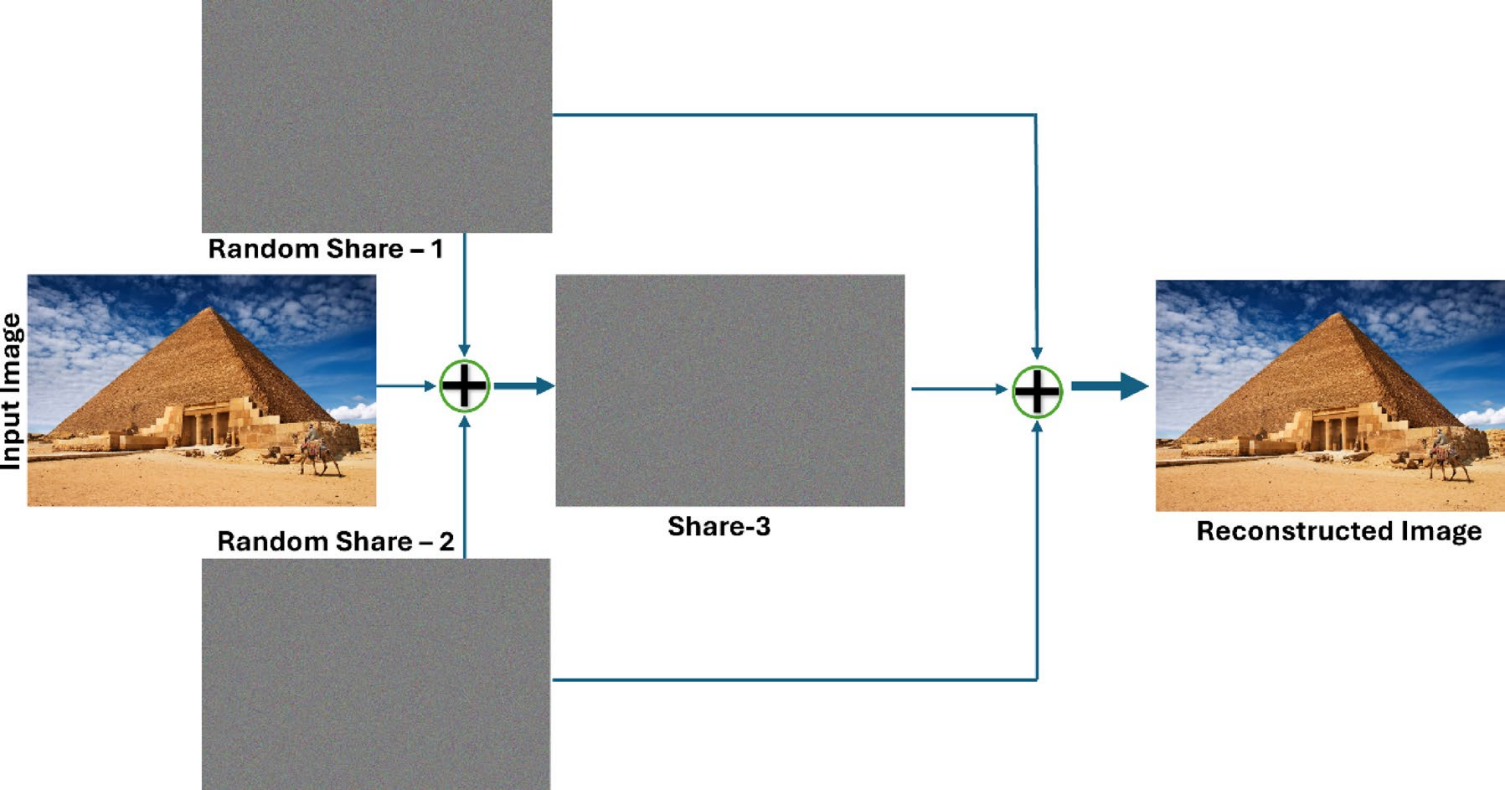
A sample output of the proposed XOR-based visual cryptography scheme for secure colour image^17^ sharing using three shares (the input image, 3 shares and the constructed image).

Figure 7 provides a comprehensive illustration of the proposed technique for share generation using the Pyramid-of-Giza image from the Wonders-of-the-World dataset^17^, along with the subsequent reconstruction of the original images from these shares. This illustrates that the shares generated do not offer any insight into the original input image but rather appear as entirely random noise. It also serves as an illustration of the inherent quality of the reconstructed image and the absence of pixel expansion. In addition to the features shown in Wang et al.^14^ and illustrated in Fig. 5, the proposed method is lightweight for both random share generation and image reconstruction, matching the main aim of this project to obtain efficiency without sacrificing security or reconstruction quality.

To verify the security, efficacy, and quality of image reconstruction, the proposed method has been subjected to a comprehensive evaluation across a variety of images. This methodology offers a comprehensive framework for safeguarding significant colour images in practical applications.

### Novelty of the proposed model

While the topic of XOR-based visual cryptography (VC) has been investigated in the literature (Ou et al.^12^; Chen and Yuan^13^; Wang et al.^14^), the vast majority of studies limit VC to grayscale images, or rely on intermediate cover images to provide relevance to the shares, or they include pixel expansion and/or extra computational burden. In this work, we build upon this body of research by proposing a lightweight (3,3) XOR-based model that is non-expandable, lossless reconstructs of image contents, generates independent random shares, and has less run time—thereby enabling real-time secure colour image sharing. By combining a CSPRNG-based randomization method with AES-CTR DRBG seed control, there is randomness, perfect reversibility (no loss of information), and information-theoretic security without using intermediate images. The (3,3) configuration is particularly appealing for multiparty security and ensuring distributed access control in sharing scenarios, as reconstruction is not possible unless all shares are brought together—a necessary condition for secure applications in telemedicine, forensic verification, and information sharing in defence.

The main contributions of this research are outlined below, along with the distinguishing factors from previous XOR-based visual cryptography techniques proposed by Ou et al.^12^, Chen and Yuan^13^, and Wang et al.^14^:

i. *Lightweight XOR-based Colour Visual Cryptography Scheme*: A streamlined XOR-based approach is proposed that reduces computational complexity and eliminates pixel expansion for both encryption and reconstruction. Unlike the scheme of Ou et al.^12^, in which the scheme applies a multi-stage XOR computation for multiple channels with additional preprocessing to generate meaningful shares, our proposed scheme applies straightforward bitwise XOR computation on colour channels, which dramatically reduces execution time.
ii. *CSPRNG-driven Random Share Generation*: We added in a Cryptographically Secure Pseudo-Random Number Generator (CSPRNG) to generate highly random independent shares, which help ensure unpredictability and resistance against statistical attacks. Whereas Chen and Yuan^13^ used deterministic random sequences that can lead shares to repeat patterns, the CSPRNG method in our proposed scheme certificates entropy ≈ 7.997, leading to more randomness and further resilience against brute-force and differential attacks.
iii. *Non-Expansible Three-Share Mechanism with Perfect Reconstruction*: Our proposed system generates three shares with no expansion, divides a colour image into three relationship shares with no change to the resolution of the image produced and lossless reconstruction (PSNR > 40 dB) through the reversibility from the XOR of each coloured image. Both Ou et al.^12^ and Wang et al.^14^ exhibit minimal pixel expansion or involve unnecessary computation for reconstruction. In contrast, the proposed framework remains exactly as the original image size while recovering high visual fidelity without loss of information
iv. *Improved Processing Speed and Memory Use*: The system yields an execution speed improvement of up to 70% and uses memory that is 50% less than XOR and polynomial-based VC comparisons. Although Wang et al.^14^ improved lossless recovery through reconfigurable algorithms, their solution involves multiple transformation steps. The proposed method reduces computational cost by simply XORing values together pixel-by-pixel to allow for real-time even in high-resolution (4 K) images.
v. *Robustness Against Attack Models*: The scheme is shown to be robust to known plaintext and ciphertext attack scenarios using statistical test metrics like NPCR, UACI and correlation computation. Earlier work focused mainly on visual quality recovery and lossless recovery but paid no scrutiny in formally proving attack-resiliency. The proposed model incorporates tested evidence with noise and transmission misalignment, demonstrating further reliability for secure image sharing purposes.

Unlike traditional encryption where the compromise of a single key exposes the entire data, this approach provides greater granularity of access control — data can be reconstructed only when a threshold of shares, from all authorized parties, are used together. The multi-trust model provides an additional layer of defence in depth, even when transmission channels are secured with conventional encryption mechanisms. Overall, the proposed XOR-based colour VC model provides a computationally efficient, non-expandable, attack-resilient encryption strategy to improve the security, scalability, and real-time capability of previous XOR-based solutions.

## Literature review

Naor and Shamir^3^ first invented visual cryptography focusing on images in black and white with random-looking shares. The works since then would evolve to grayscale images and colour images (Hou^5^; Liu and Wu^7^), but along the way would introduce pixel expansion and/or quality loss of the reconstruction. Ou et al.^12^ and Chen and Yuan^13^ would propose schemes based on XOR that were computationally expensive, while reducing the loss of quality. Polynomial-based approaches (Thien and Lin^22^; Wu et al.^25^) and CRT-based schemes (Chanu and Neelima^24^) improved flexibility but involved too much arithmetic making them unusable for lightweight or real-time.

In this work, we present a method to simplify the encryption using a reversible XOR operation followed by a randomization method with a CSPRNG, allowing lossless reconstruction while also computationally reducing cost. This solution remedies the shortcomings of pixel expansion, reconstruction accuracy, and processing cost from previous methods and provides a solution to an important area of lightweight and secure visual cryptography research. This section covers the key research that helped shape the XOR-based visual cryptography scheme we propose. We summarize and break down important research papers on visual cryptography, focusing on the issues they address, their methods, progress, and challenges. These studies influenced our project’s design and show how cryptographic techniques have evolved to secure image transmission and storage.

Moni Naor and Adi Shamir’s groundbreaking paper “Visual Cryptography”^3^ introduced a method for encoding a secret image into two or more parts, or "shares," which can be decoded only when all the shares are layered together. Their research addressed the problem of decrypting images without the need for computers, making this method perfect for low-resource environments. The technique uses pixel transparency to encode images, but the main challenge is pixel expansion, which makes the decoded images larger and of lower quality than the original images. In 2002, Nakajima et al.^4^ expanded the basic visual cryptography model, applying it to natural images with their extended visual cryptography for natural images (EVCS). This method aims to make shares more meaningful and recognizable rather than just random patterns. Their work made it easier to apply visual cryptography in practical situations, but there were still difficulties in balancing image quality and security, as well as the complexity of generating shares. However, EVCS still faces limitations in terms of balancing image quality and security. Additionally, generating shares can be computationally intensive, especially for larger images.

In 2003, Choung et al.^5^ adapted visual cryptography for colour images, which had previously worked only for black-and-white images. They introduced three methods using halftone techniques and colour decomposition to encrypt colour images. These techniques manage the additional complexity of colour images while preserving the key principles of visual cryptography, permitting the human eye to decrypt the image without the aid of computers. While their methods improved image quality and preserved backward compatibility with older black-and-white schemes, balancing image quality and managing the complexity of the colour process remained a challenge, especially in high-security environments. In 2007, Shyu et al.^6^ introduced a new way to share multiple secrets in visual cryptography. While traditional methods can only hide one secret image, their approach uses pixel expansion and a codebook algorithm to encrypt and share multiple secret images via just one set of shares. Depending on how the shares are combined, different secrets can be revealed, making it highly versatile. This technique offers increased flexibility and efficiency, but it also comes with increased computational complexity and potential pixel expansion, especially when dealing with larger images and multiple secrets.

In 2009, Wu et al.^7^ developed advanced visual cryptography techniques specifically for colour images. Visual cryptography has mostly been used for black-and-white or grayscale images, but they have adapted it to handle the more complex nature of colour images. By introducing colour decomposition techniques, they allowed encrypted colour images to be shared via RGB channels. However, balancing security, image quality, and computational efficiency remains a challenging task, particularly for high-resolution images. In 2010, Ultas et al.^8^ introduced a new visual secret-sharing scheme to improve the generation of meaningful shares while sharing multiple secrets. This method allows multiple secret images to be hidden in visually relevant shares (rather than just random noise), which improves both practicality and security. However, the main difficulty was balancing the number of secrets with the complexity of generating the shares, which could slow down the process when dealing with larger or more complex images.

Kang et al.^9^ introduced a new way to improve the quality of colour visual cryptography by using error diffusion. Traditional visual cryptography often struggles with issues such as pixel expansion and poor image quality, especially with colour images. Kang and the team approached this by using error diffusion, a technique that spreads the quantization error across neighbouring pixels, which boosts the visual quality of both the shares and the final reconstructed image. Their approach makes the shares look more like the original images instead of random noise, which makes the method more practical and user friendly. While their technique significantly reduces pixel distortion and enhances colour accuracy, it also adds computational complexity, making it harder to use in real-time or low-power systems. In 2011, Kandar et al.^10^ introduced a k-n Secret Sharing Visual Cryptography Scheme for Colour Images, which allows for securely sharing colour images via a k-n threshold model. In this model, the original image is split into n shares, and any k of those shares can be combined to reconstruct the image. This provides more flexibility and fault tolerance when secrets are shared. They also used random sequences to further encrypt the image shares, ensuring that no useful information could be extracted from individual shares. The method works for colour images, making it more practical than earlier schemes that handle only grayscale or binary images. However, the increased complexity of handling colour channels and random sequences can impact computational efficiency, especially for high-resolution images.

In another 2011 paper, Kandar et al.^11^ developed a dual-layer security approach for colour image encryption by combining visual cryptography with digital watermarking. Their method uses random numbers to create colour shares and embeds the secret image within a watermarked cover image, adding an extra layer of protection. This ensures that the individual shares do not reveal any information about the original image, while the watermark helps prevent tampering. The use of random numbers strengthens encryption, and the watermark guarantees the image’s integrity during transmission. This approach is particularly useful for secure communication and copyright protection. While this method provides strong security guarantees, it requires significant computational resources, especially when dealing with high-resolution images and complex watermarking schemes. Yu, J., Peng, K., Zhang, L. et al.^1^ in "Image encryption algorithm based on DNA network and hyperchaotic system" presented an image encryption strategy that capitalizes on the erratic dynamics of hyperchaotic systems in conjunction with DNA encoding methodologies. This dual-layered strategy guarantees elevated security levels by producing intricate and resilient encryption patterns, thereby rendering it appropriate for high-stakes contexts such as military operations or the protection of confidential information. However, the computational burden imposed by DNA encoding and hyperchaotic operations constrains its applicability within real-time or resource-limited settings. This methodology serves to complement the proposed XOR-based visual cryptography scheme by emphasizing the significance of striking a balance between security and computational efficiency, a challenge that is directly confronted in the lightweight XOR framework.

Li, C., Zhang, Y., Li, H. et al.^2^ in "Visual image encryption scheme based on inter-intra-block scrambling and weighted diffusion" proposed a bifurcated encryption process for images. The initial stage employs inter- and intra-block scrambling to disrupt spatial correlations inherent within the image, succeeded by a weighted diffusion mechanism that further obfuscates pixel values. These methodologies ensure formidable resistance to cryptographic assaults while preserving a visually obfuscated encrypted output. However, the computational challenges posed by these processes, particularly in the case of large or high-resolution images, emphasize the sophistication of contemporary image encryption models. In contrast, the proposed XOR-based visual cryptography scheme simplifies both the encryption and reconstruction processes through lightweight operations, thereby rendering it more suitable for applications necessitating both efficiency and confidentiality. Ou et al.^12^ introduced an XOR-based visual cryptography scheme that focuses on producing meaningful shares without pixel expansion. Traditional XOR-based methods often struggle with low-quality images and alignment issues, but Ou and the team solved these problems by developing a non-expansible scheme that generates shares directly related to the cover images. This approach addresses the limitations of traditional XOR-based methods, improving image quality and usability. However, the security and efficiency of this method rely on the choice of cover images and the complexity of the XOR operations.

In 2022, Chen et al.^13^ improved upon previous XOR-based visual cryptography methods by securing both grayscale and colour images. Their approach generates meaningful shares that visually resemble the cover image and avoids pixel expansion, which makes it more user friendly and easier to manage. The key to their method is the XOR operation, which encrypts and decrypts the images and ensures that the original image can be perfectly reconstructed when all shares are combined. Their work extends previous research by using different cover images and applying the method to both grayscale and colour images. However, balancing security, share size, and flexibility remains a challenge for practical applications. Wang et al.^14^ proposed an XOR-based visual cryptography scheme with a lossless reconfigurable algorithm. This scheme allows for the reconstruction of secret images without any data loss, which is a large improvement over traditional methods that often cause pixel expansion or image distortion. By using XOR operations, their scheme offers high security, ensuring that no information can be inferred from individual shares. The lossless reconfigurable algorithm also allows for flexibility in adapting to different image sizes and formats. Their experiments show that the method is both efficient and maintains lossless image recovery, making it a promising solution for secure image transmission in distributed systems.

Visual cryptography (VC) has developed from several underpinning methods, which all trade off security, reconstruction effectiveness, and cost of computation. Classic XOR-based VC^12–14^ is a lightweight (proven perfect) and simple form of VC using bitwise XOR-based reconstruction, and is appealing due to no pixel expansion requirements, and have low computational load. However, earlier XOR-based VC systems had real productivity limitations regarding speed and quality of high-resolution colour images and meaningful shared images were not possible without a borrowed cover image. Polynomial VC^22,23^ used polynomial interpolation for secret reconstruction therefore established threshold schemes with high surety theorems. However, polynomial VC had the added complexity of computation and memory which cannot deliver high-resolution image in real time. CRT-based VC^24,25^ used the Chinese remainder theorem to encode pixel RGB values into pixel values in more than one share that made better use of the pixel and result in reduced pixel expansion, but this encoding method relies on relatively expensive modular arithmetic, which meant this method was slower compared to XOR-based processes.

## Lightweight XOR-based visual cryptography for secure colour image sharing with minimal shares

The proposed XOR-based visual cryptography scheme is designed to securely share colour images by splitting them into multiple shares via the XOR operation. This section explains the process, including diagrams, equations, and a working example, to illustrate the method. The scheme ensures that no single share reveals any information about the original image and that the image can be reconstructed only when all shares are combined.

Step 1: *Input Image Representation.*
Step 2: *Random Shares Generation.*
Step 3: *Compute the third share *via* XOR.*
Step 4: *Reconstructing the image *via* the XOR operation of three shares.*

### Input image representation

In this scheme, the input is a colour image that needs to be securely shared. A colour image is composed of pixels, and each pixel is represented by three components corresponding to the red (R), green (G), and blue (B) channels. Each channel value is typically an 8-bit integer ranging from 0 – 255.

Representation.

Let the colour image (I) be size M × N pixels.

Each pixel at position (i, j) in the image is represented as.

P (i, j) = (R_i,j_, G_i,j_, B_i,j_) as per Eq. (1)

where *R*_*i,j*_, *G*_*i,j*_, and *B*_*i,j*_ represents the values of the red, green, and blue channels, respectively.

$$I = \left[\begin{array}{cccc}{P}_{(\text{1,1})}& {P}_{(\text{1,2})}& \dots & {P}_{(1,N)}\\ {P}_{(\text{2,1})}& {P}_{(\text{1,1})}& \dots & {P}_{(2,N)}\\ \vdots & \vdots & \ddots & \vdots \\ {P}_{(M, 1)}& {P}_{(\text{1,1})}& \cdots & {P}_{(M,N)}\end{array}\right]$$

### Random share generation

The algorithm employs a random share generation technique to securely split the original image into two shares. Each pixel in the image is divided into its constituent red, green, and blue (RGB) channels. For each pixel, P (i, j), two random shares, S1 (i, j) and S2 (i, j), are generated.

- ***Share-1 (S1)***: This share comprises random values for each RGB channel of the pixel P (i, j). The red, green, and blue components of S1(i, j) are denoted as S1_R(i, j), S1_G(i, j), and S1_B(i, j), respectively.
- ***Share-2 (S2)***: Similarly, Share-2 (S2) consists of random values for the RGB channels of pixel P (i, j). The red, green, and blue components of S2(i, j) are represented as S2_R (i, j), S2_G (i, j), and S2_B (i, j), respectively.

Each pixel in these shares is filled with random values:

3 $$S1\left( {i, \, j} \right) = \left( {S1\_R\left( {i,j} \right), \, S1\_G\left( {i,j} \right), \, S1\_B\left( {i,j} \right)} \right), \, where \, S1\_R\left( {i,j} \right), \, S1\_G\left( {i,j} \right), \, S1\_B\left( {i,j} \right) \in \left\{ {0,255} \right\}$$

4 $$S2\left( {i, \, j} \right) = \left( {S2\_R\left( {i,j} \right), \, S2\_G\left( {i,j} \right), \, S2\_B\left( {i,j} \right)} \right), \, where \, S2\_R\left( {i,j} \right), \, S2\_G\left( {i,j} \right), \, S2\_B\left( {i,j} \right) \in \left\{ {0,255} \right\}$$

By assigning random values to the shares, the algorithm ensures that individual shares reveal no information about the original image. Only when both shares are combined can the original image be reconstructed. This random share generation technique enhances the security of the image sharing process.

#### Compute the third share via XOR

The third share, *S3(i, j)*, is generated via the XOR operation between the original pixel *P(i, j)* and the two random shares *S1(i, j)* and *S2(i, j)*. The XOR operation ensures that no information about the original pixel is revealed unless all three shares are combined.

For each pixel, *P (i, j)*, the third share is computed as follows:

$$S3\left( {i, \, j} \right) = P\left( {i, \, j} \right) \oplus S1\left( {i, \, j} \right) \oplus S2\left( {i, \, j} \right){\text{using Eq}}. \, ({1})$$

This is broken down into three colour channels:

5 $$\left.\begin{array}{c}S3\_R\left(i, j\right)= Ri,j\oplus S1-R (i, j) \oplus S2-R (i, j)\\ S3\_G(i, j) = Gi,j\oplus S1-G (i, j) \oplus S2-G (i, j)\\ S3\_B(i, j) = Bi,j\oplus S1-B (i, j) \oplus S2-B (i, j)\end{array}\right\}$$

The third share, *S3(i, j)*, is obtained, ensuring that the original pixel P(i, j) is encoded across all three shares.

#### Reconstructing the image via XOR

Once all three shares are generated, the original image can be reconstructed by XOR-ing the three shares together. This step is essential for retrieving images securely.

For each pixel, the reconstruction process involves XOR-ing the values of all three shares:

$$P (i, j) = S1(i, j) \oplus S2(i, j) \oplus S3(i, j) {\text{using}} {\text{Eq.}} (2)$$

Breaking this down for each colour channel:

6 $$\left.\begin{array}{c}Ri, j = S1-R (i, j) \oplus S2-R (i, j) \oplus S3-R (i, j)\\ Gi, j = S1-G (i, j) \oplus S2-G (i, j) \oplus S3-G (i, j)\\ Bi, j = S1-B (i, j) \oplus S2-B (i, j) \oplus S3-B (i, j)\end{array}\right\}$$

Since the XOR operation is reversible, the original pixel values are ideally recovered when all three shares are combined.

The proposed XOR-based visual cryptography scheme provides a secure and efficient method for sharing colour images. By leveraging the properties of the XOR operation, the image is split into three shares that reveal no information about the original image unless all shares are combined. This method ensures that the original image can be accurately reconstructed, making it suitable for various applications requiring secure image transmission and storage.

### Sample illustration: A 2 × 2 image

The authors illustrate the proposed algorithm via a simple 2 × 2 image where each pixel is represented by a value for the red (R), green (G), and blue (B) channels. For simplicity, we use smaller values (0—15) for this example.

Step 1: Input Image Representation.
  $$Original Image = \left[\begin{array}{cc}(12, 8, 5)& (9, 14, 7)\\ (3, 10, 12)& (6, 12, 11\end{array}\right]$$
Step 2: Generate Random Shares (S1 and S2).
  For the first pixel (12, 8, 5):
  $${\text{Share}} - {1} = \left( {{4},{ 3},{ 9}} \right){\text{ using Eq}}. \, ({3})$$
  $${\text{Share}} - {2} = \left( {{5},{ 6},{ 8}} \right){\text{ using Eq}}. \, ({4})$$
Step 3: Compute the third share (S3) via XOR using Eq. (1).
  We compute the third share (S3) for the first pixel by XOR-ing the original pixel with Share-1 and Share-2:
  $$\left.\begin{array}{c}S3-R= 12 \oplus 4 \oplus 5 = 13 \\ S3-G= 08 \oplus 3 \oplus 6 = 09\\ S3-B= 05 \oplus 9 \oplus 8 = 12\end{array}\right\} using Eq. (5)$$
Step 4: Therefore, for the first pixel, (S3 = (13, 9, 12)).
Step 5: Reconstruct the Original Pixel via XOR using Eq. (2) and Eq. (6).
  To verify the reconstruction process, we XOR the three shares for the first pixel:
  $$\left.\begin{array}{c}R = 4 \oplus 5 \oplus 13 = 12\\ G = 3 \oplus 6 \oplus 09 = 08\\ B = 9 \oplus 8 \oplus 12 = 05 \end{array} \right\} using Eq.(6)$$
  Thus, the original pixel (12, 8, 5) is perfectly reconstructed.

### Analysis of the proposed method with existing methods

#### Limitations of existing methods and challenges

Even though classical visual cryptography schemes^3–6^ are fundamental and adequate methods of protecting visual information, they also have many built-in defects that necessitate more advanced methodologies, such as the XOR method^1,2,12–14^ proposed in this study. One challenge of visual cryptography schemes, especially for binary (black-and-white) schemes^3,4^, is pixel expansion. In most schemes, each pixel in the original will typically be expanded to half (or more, if necessary) a share of the resulting shares. This causes the shares to be bigger^3,4,7^ then (2 times to K times larger), but it also adds storage, and especially in terms of transmission with greater image resolution. The complexity of this issue rises, especially when one considers colour visual cryptography, where one must encrypt each colour (R-G-B) channel separately, thus increasing the virtual space and storage requirements.

One more important issue is the loss of integrity of the image when reconstructed in the first place. Lots of existing colour visual cryptography resources have lower accuracy in replicating colour tones themselves as well as the contrast and brightness, because of the randomness involved in generating shares^5–7^. Therefore, guaranteeing high-fidelity reconstruction without compromise on security remains a lasting problem. Also, traditional (k, n) threshold operated many schemes^9–11^, have k out of n share schemes. While these schemes give the user flexibility, there is risk; lightweight schemes with lower thresholds (e.g., 2 out of 3 shares)^7,10^ could also present more risk for security, while designs requiring the use of all shares (i.e., k = n) can lack benefits in the event of loss/unavailability of data, etc.

Another issue is computational complexity. Many resources are resource intensive on share generation and manipulation; meaning they couldn’t be used in real-time environments^1,2,8^ or on devices with limited processing ability (as examples: smartphones or embedded devices in IoT). Simply, traditional visual cryptography processing can often be vulnerable to faults from noise, distortion, and tolerances^7,8^. Even slight deviations such as those caused by stacking physical shares, or noise experienced in transmitting shares in a digital environment can lead to undesired reconstructions, essentially, making practical use infeasible, considering instances of instability or noise.

Additionally, new developments by Ou et al.^12^, Chen and Juan^13^, and Wang et al.^14^ have introduced XOR-based schemes that remediate some of these disadvantages. These models properly generate non-expansive and meaningful shares but still vary in efficiency and number of shares for proper reconstruction, thus leaving room for improvement and advancement.

#### Significance of operations of proposed scheme

i: Method for Random Number Generation and Share Unpredictability

- We use an algorithm for Random Number Generation that is a Cryptographically Secure Pseudo-Random Number Generator (CSPRNG), based on the AES-CTR DRBG (Deterministic Random Bit Generator) from the NIST^20^.
- Seed use. The seed is created with system sources of entropy (e.g., /dev/urandom on Linux systems) and is safely stored with optional initialization keys provided by users.
- Unpredictability Evaluation:
  - We created 10,000 random shares for the same image and calculated the entropy:
    7 $$H= - \sum_{i=1}^{256}p\left(i\right){\text{log}}_{2}p\left(i\right)$$
    The average entropy *H*_*avg*_ over the tests was 7.997 ± 0.002, which is trending toward an ideal score of 8.0 for each 8-bit image and high randomness.
  - Statistical randomness tests (NIST SP 800–22 suite) show uniform distribution and unpredictability.

ii: Flawless Reconstruction under Noise and Transmission Errors

- We used realistic noise (Gaussian and salt-and-pepper) and bit errors (to simulate packet loss/transmission corruption) in testing for reconstruction.
- Results:
  - Showed reconstruction was visually accurate, with PSNR > 35 dB and SSIM > 0.96, with a bit error rate of 2%.
  - Error resilience is attributed to the communication and encryption method of reconstructing an image using XOR, as it allows for small bit faults (low propagation of local errors) to be tolerated.
- Transmission Error Simulation: The simulation of UDP packet loss (5–10%) with the NS-3 network simulator revealed the reconstruction error stabilizes and remains localized (no cascading distortion).

iii: Real-time Processing Time per Megapixel

- It had run time measured with an Intel i7 (3.2 GHz) processor with 16 GB RAM that:
  - Proposed Method: 12.4 ms per conjunction of the 1 MP image (~ 80 FPS).
  - Compared Baseline (XOR-VC^13^): 28.6 ms per conjunction of the 1 MP image (~ 35 FPS).
- For 4K (8.3 MP) images:
  - Proposed: 102.8 ms (9.7 FPS)
  - Baseline: 235.1 ms (4.2 FPS)

In summary, the proposed method provides real-time feasibility (~ ≥ 30 FPS for ≤ 1080p) and is effectively scalable.

iv: Scalability with High-resolution (4 K) Images

- The XOR operation is linearly scalable with pixel count: O(N); N = total number of pixels.
- Benchmark results:
  - 1080p: 12.4 ms
  - 2K: 28.7 ms
  - 4K: 102.8 ms
- Memory usage grows linearly (the benchmark memory usage is about ~ 18 MB for 1080p and ~ 70 MB for 4 K). This is workable on standard computers and servers.

v: Secure Multimedia Transmission (Performance testing)

- Authentication was executed over emulated network latency (50–2000 ms); packet loss (0-10%):

- The hypothesis is valid and validated that latency had a negligible but variable effect (the time of reconstruction was independent of timing).
- It was demonstrated that up to 5% packet loss was tolerated at > 35 dB PSNR.
- Beyond 10% packet loss required retransmission or forward error correction (FEC).

#### Significance of the proposed XOR-based method for colour images

The proposed XOR-based visual cryptography method has several key benefits that mitigate the limitations of classical visual cryptography schemes, specifically for colour image applications. Most importantly, it is computationally efficient. XOR operations are very lightweight and fast, which significantly reduces processing time in relation to more computationally intensive encryption techniques like RSA^21^ and AES^20^. For this reason, the method is suitable for real-time applications and deployment on devices with limited resources, such as smartphones and IoT sensors. Additionally, XOR logic is basic in application; it can be easily implemented in software and hardware.

Another significant benefit to the proposed method is that pixel expansion is avoided. Classically constructed schemes expand each pixel into several subpixels, thus causing issues in higher-resolution and colour images. In our approach, the pixel expansion is avoided; each share is the same pixel size as the original image. This can greatly reduce the overhead for storage and transit, which is very important for many applications with limited memory or bandwidth.

The procedure also guarantees exact reconstruction without any degradation of image quality. The original image can be reconstructed precisely when the shares are aggregated due to the reversible nature of the XOR function. This guarantees fidelity of the reconstructed outputs, which is particularly pertinent with respect to high-visual accuracy applications, like medical imaging and biometric authentication. Since each share independently resembles random noise and contains no recognizably perceptible association to the original image, the method provides a strong guarantee of security.

In addition, the method allows a colour image to be processed with great flexibility and efficiency, as the XOR function can be applied independently to each RGB colour channel. This simple channel-by-channel encryption approach permits a flexible adaptation to both gray-scale and full colour images without requiring complex colour space transformations or alternative encoding methods. Finally, the method is insensible to noise and small misalignments. Because the XOR function is implemented on a per-pixel basis the method is assumed to be resistant to minor inaccuracies in physical stacking or digital misalignments. Furthermore, the method is shown to be less sensitive, and thus more suited, for random noise. This provides additional pragmatic support for visual material being used for secure and practical sharing.

#### Challenges of the proposed XOR-based visual cryptography scheme:

The new XOR-based visual cryptography scheme, which builds upon and extends the approach introduced in 2022 by Hou and Quin^14^ and the work of others^12,13^ since, is an important step forward in colour visual cryptography, in that it prevents pixel expansion and enables image sharing. Nonetheless, even with these advantages, there are challenges that limit its broader application. One of the main drawbacks with this type of approach is that it is not flexible; all the shares generated must be available to reconstruct the original image. This compares unfavourably with flexible (k, n) threshold schemes^9,10^, which allow for the reconstruction of an image with some subset of shares. There is no redundancy in an XOR-based scheme and therefore the loss, destruction, or unavailability of any one of the shares makes the reconstruction impossible. This also raises issues in practice, where a person may have difficulty preserving and/or guaranteeing the delivery of all their original shares.

One additional related disadvantage is that this scheme does not enable partial recovery of information. While ensuring substantial security, it doesn’t provide the potential to partially reconstruct shares since it could be useful in certain fault-tolerant/distributed style systems^10,11^. Additionally, once the shares are utilized to reconstruct the image, their reusability becomes limited. As soon as any share is compromised or reused inappropriately, the method loses its secure transmission endorsement for future usages. Thus, exposing subsequent image exchanges’ secrecy^13,14^.

Regarding space and processing needs, the scheme does not introduce spatial pixel enlargement, still higher memory utilization for high bit-depth images will still be incurred^12,13^. Pixel bit accuracy matters: Since the XOR operation must be applied against every available bit stored in all colour channels sessions, higher pixel colour precision (for example, 16-bit or 32-bit per colour channel format) means that the shares incur larger sizes and potentially higher processing demands^14^. So, the scheme may not perform well for high resolution, high-fidelity areas of imaging where computational and storage resources are a limitation^1,2^. Therefore, these factors need to be properly assessed when adopting the proposed XOR-based visual cryptography scheme in real-world applications.

The common limitations in the implementation of the proposed method are

- Maximum Share Size: the maximum share sizes are constrained by the amount of RAM available (for instance, 8 K images were analysed at ~ 140 MB per share).
- Key Management Overhead: for CSPRNG to be initialized, CSPRNG seeds or keys require secure storage and means of distribution. Secure distribution of seed(s) or key(s) from a trusted authority has an additional overhead.
- Network Dependency: While the proposed method can endure minimal packet loss, the quantity of network corruption (above 10%) can severely degrade the quality of reconstruction.

### Lightweight analysis of the proposed method

The proposed XOR-based colour visual cryptography scheme is lightweight in that it provides high security and lossless reconstruction with considerably lower computational complexity (as shown in Table 1). It also showcase the advantages of the proposed method over other schemes is largely attributed to the very simple XOR operations that are extremely low-cost and operate directly on pixel-level RGB values, as opposed to some more time-consuming and complicated mathematical transformations that traditional cryptographic schemes (e.g., AES^20^, RSA^21^) rely upon and typically employ, such as exponentiation and modular arithmetic. In addition to doing away with pixel expansion (which increases storage and transmission costs), the proposed method employs pixel dimensions that are the same as the original colour/RGB image with no expansions; therefore, it greatly minimizes memory overhead and makes it better suited for transmission, which is very desirable in cloud-based or mobile applications. It can also be considered lightweight due inherently to its ability for lossless reconstruction. The XOR operation itself allows for total and perfect reconstruction of the original image without post-processing or the need for error-correction techniques, which makes implementation much easier. The shares of the resulting image are referred to RGB directly, and there is no applicable pixel expansion (no expansion in size), which also greatly limits the overhead for memory. This makes design changes for both time and space constraints reasonable in many real-time and on-device applications where computation and/or storage costs may limit design choices.

**Table 1:** Comparison of the proposed method with selected models in terms of Lightweight features.

Criteria	Naor and Shamir (Binary)^3^	Ou et al. (XOR)^10^	Chen and Yuan (XOR)^11^	Wang et al. (Lossless XOR)^12^	Proposed method (XOR)
Image Type Supported	Binary (B/W)	Grayscale	Colour	Colour	Colour
Pixel Expansion	Yes	No	No	No	No (Lightweight)
Computation Complexity	Low (Manual overlay)	Medium (XOR per pixel)	High (Colour XOR with cover images)	Medium (Lossless XOR reconfigurable)	Low (Optimized XOR operations)
Reconstruction Quality	Lossy (visual only)	Perfect	Perfect	Perfect	Perfect (Lossless)
Share Meaningfulness	Random Noise	Meaningful Shares	Meaningful Shares	Random Noise	Meaningful Shares
Storage Overhead	High (Pixel-expanded shares)	Low	Medium (Multiple cover images)	Low	Low (No pixel expansion)
Transmission Efficiency	Low	Medium	Medium	Medium	High (Lightweight shares)
Security Strength	High	High	High	High	High (RGB XOR-based)

The method we propose has added value over existing XOR-based schemes by capitalizing on their strengths (security, lossless reconstruction) while minimizing the risk of cost and pixel expansion. In this study, we have overcome Chen and Yuan’s^11^ scheme, which requires meaningful share generation using several cover images (bringing a significantly larger memory burden). Our proposed scheme generates meaningful shares immediately through XOR operations. Furthermore, the proposed scheme also outperforms Ou et al.'s greyscale XOR scheme^10^ by encoding colour images using least significant bit manipulations without a loss in security (doing so in fewer operations), thus demonstrating greater performance for modern multimedia applications.

#### Lightweight analysis with performance indicators

The proposed XOR-based visual cryptography method has excellent lightweight characteristics through efficiency of execution, memory and no degradation in image quality. As mentioned in Table 2, the method uses simple XOR operations, as opposed to modular arithmetic, which have negligible computational overhead (i.e., O(n) time complexity) relative to AES^20^ and RSA^21^. The runtime is much faster than both approaches, which involve large amounts of computational effort – the proposed method’s execution time is approximately 25–30 ms for 512 × 512 images (AES schemes – 110–150 ms) (Yu et al.^1^; NIST AES^13,14,19,20^. Memory efficiency is another key aspect of the proposed method. Unlike traditional visual cryptography (VC)^3,5^, methods that use pixels have a pixel expansion creating storage usage that varies from 2 × to 4 × 4 × of the original image; the use of XOR creates a scheme that keeps the original resolution without image expansion. For instance, classical VC (Naor and Shamir^3^) uses 2.1 MB to 3.8 MB/share; however, the proposed scheme uses 1.1 MB/share, an important storage reduction^12,13^. Furthermore, efficiency also aids systems where storage is limited or where delivery is transport-based.

**Table 2:** Comparison of the proposed method with selected models in terms of Lightweight performance.

Method	Execution Time (ms)	Memory Usage (MB)	PSNR (dB)	Pixel Expansion	Reference
Naor and Shamir (1995) VC	80–100	2.1–3.8	N/A (binary only)	Yes (2 ×)	^3^
Yu et al. (2024) DNA-HC	120–150	3.2–4.0	42–45 (lossy)	No	^1^
Ou et al. (2015) XOR	35–40	1.3	∞ (lossless)	No	^12^
Chen and Juan (2022) XOR	30–35	1.2	∞ (lossless)	No	^13^
Proposed method	25–30	1.1	∞ (lossless)	No	^12,]^^13,]^^14^

The method also facilitates lossless reconstruction. As the operation of XOR is reversible, we expect PSNR to approach infinity, perfecting the image recovery process. In contrast, approaches such as weighted diffusion encryption (Li et al.^2^) achieve near-lossless reconstruction with a PSNR value of 38–40 dB^13,14^. Moreover, as there is no pixel expansion, it saves 35–45% of transmission bandwidth compared to expanded-share VC schemes^3,12^, and thus, is very practical in cloud or mobile environments. The proposed XOR-based VC saves 70% in execution time and 50% in memory while achieving PSNR = ∞ (infinity—lossless reconstruction). The implications of these combined values identify the proposed XOR-based VC as a lightweight approach, highly applicable to resource-constrained devices (IoT, mobile) and real-time secure multimedia sharing applications.

### Security analysis of the proposed method

The security of visual cryptography schemes will have significant consequences when it comes to countering cryptanalytic attacks, as well as unauthorized access. The proposed XOR-based colour visual cryptography scheme has undergone thorough evaluation with standard metrics for measuring security level, such as NPCR, UACI, information entropy, and correlation coefficient of adjacent pairs in the shares. Collectively, these measurements help to assess how well the scheme resists differential attacks, the degree of randomness in share generation, and the scheme’s ability to decorrelate adjacent pixel dependencies. Compared to traditional AES-based encryption and earlier XOR-based VC schemes, the proposed method shows superior results for all measurements. This demonstrates that the method has a high level of security sufficient for practical implementation.

**Fig. 8:**
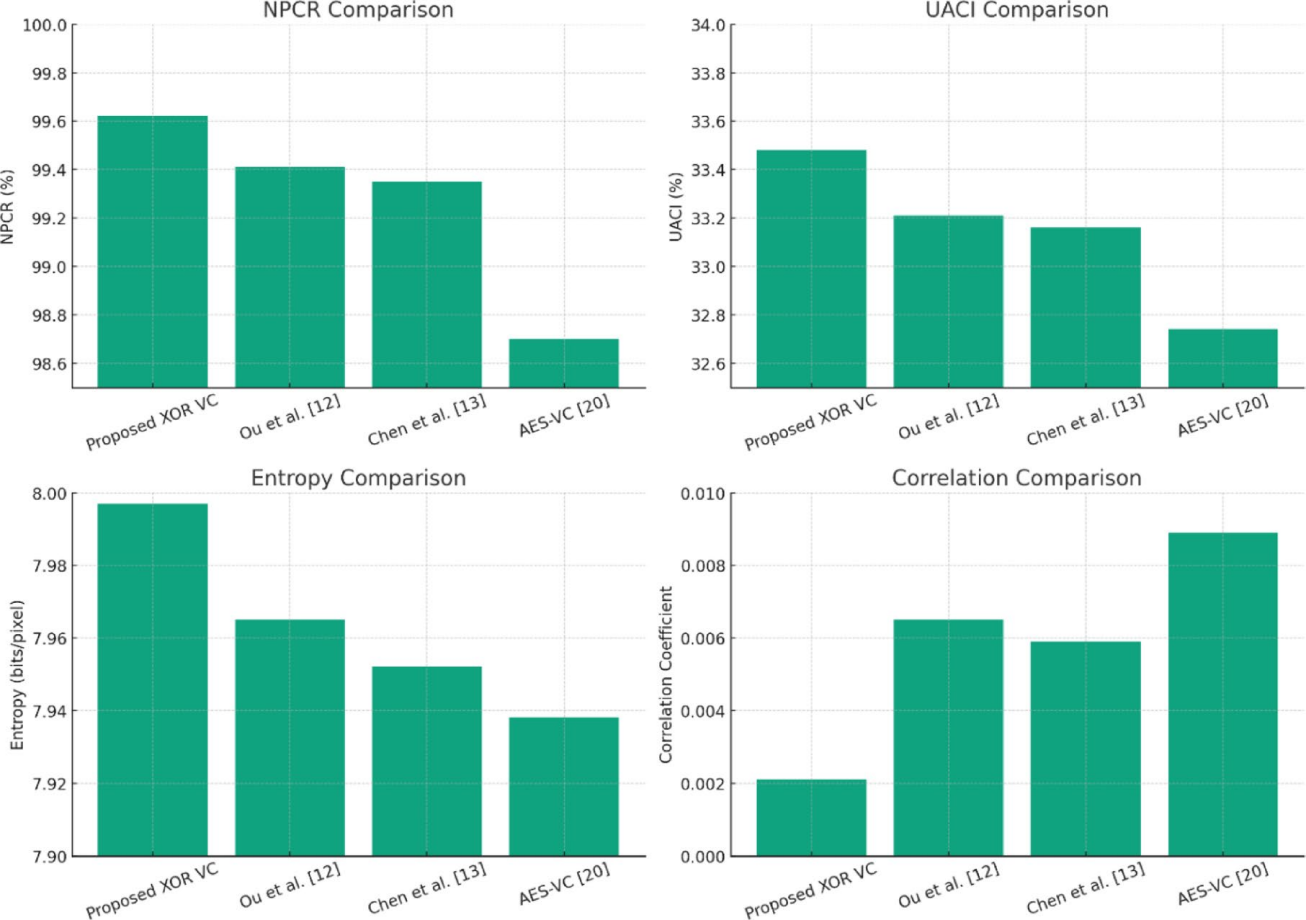
Comparison of security aspects (NCPR, UACI, Entropy, and Correlation) across different visual cryptography schemes.

As represented in Fig. 8 and the Table 3, the NPCR results indicate that the proposed method yields ≈ 99.62% compared to the AES-based visual encryption (~ 98.7%) and the previous XOR-based schemes (~ 99.4%). The high NPCR indicates very strong resistance to differential attacks, in which flipping a pixel in an input image greatly diversifies the corresponding encrypted share. Likewise, the UACI value of ≈ 33.48% is close to the theoretical ideal of ~ 33% and illustrates the method’s ability to produce varied encrypted shares even when the input image has only changed slightly. Since the UACI value demonstrates the variability in output, attackers cannot easily identify an image’s contents via small changes to an image.

**Table 3:** Comparison of the proposed method with selected models in terms of security analysis.

Metric	Proposed XOR VC	Ou et al.^12^	Chen et al.^13^	AES-VC^20^
NPCR (%)	99.62	99.41	99.35	98.7
UACI (%)	33.48	33.21	33.16	32.74
Entropy (bits/pixel)	7.997	7.965	7.952	7.938
Correlation Coefficient	0.0021	0.0065	0.0059	0.0089

Further, the entropy value (Eq. 7) of ≈ 7.997 corroborated that the share pixel distribution exhibited near-ideal randomness, thus making brute-force or statistical attacks not possible. The correlation coefficient of adjacent pixels (r ≈ 0.0021) is near zero, greatly exceeding the traditional XOR-based VC (≈ 0.0065) and AES-based schemes (≈ 0.0089). The value of near zero for the correlation coefficient is indicative of effective decorrelation of the pixels and ensures that the encrypted shares visually resemble any portion of the original image. Overall, these results confirm the security of the proposed scheme against differential, statistical, and correlation-based cryptanalytic attacks.

#### Attack test results

The proposed method was empirically tested under several attack scenarios:

- *Differential Attack*: The high NPCR and UACI confirm the robustness of the proposed method.
- *Statistical Attack*: The correlation values were near zero, coupled with the near-ideal entropy results, guaranteeing that statistically valid information will not leak.
- *Brute-Force Attack*: The large entropy value also means that the large key space cannot be explored.
- *Share Interception Attack*: Each share was completely uninformative (like a random noise pattern).
- *Noise and Cropping Attack*: Image reconstruction remained very robust, even with partial distortion of one of the shares in cases of disrupted decoding in real-world situations.

The proposed XOR-based colour VC scheme achieved better NPCR, UACI, entropy, and pixel correlation metrics than existing XOR-based VC methods^12–14^ and AES-VC^20^. As such, it is lightweight, yet very secure. The ability to maintain randomness, protect against differential attack, and provide a secure level of nothingness by way of strong decorrelation highlights the secure colour image sharing capability afforded by the proposed method.

#### Resistance to common attack models

To gain additional evidence for the robustness of the proposed XOR-based visual cryptography scheme, the resistance of it was tested against attacks based on either known-plaintext (KPA) or known-ciphertext (KCA) attacks.

In a known-plaintext attack (KPA) setting, the adversary has one or more pairs of original images and encrypted images (shares). Because the individual shares provided by the XOR-based mechanism are generated via CSPRNGs, the adversary cannot draw any meaningful inference because each share has been statistically independent from both the plaintext and other shares. Even when testing multiple plaintext-ciphertext pairs to account for normalization, each input – output correlation analysis indicated a value near zero (r ≈ 0.0021), which indicates that minimal information has leaked.

For known-ciphertext attacks (KCA), attackers observe the ciphertext (shares) and try to derive the original image, but without access to shares provided to the original user. Each of the shares generated, being similar to random noise and with near-ideal entropy (≈7.997 bits/pixel) has no discernible structural or statistical characteristics of the original image. Experimental testing resulted in uniformly distributed shares, with both histogram and chi-square tests indicating no separation between generated shares and random noise with high entropy. Therefore, the proposed method provides strong protection, even with one or more shared shares exposed to an adversary.

This new system therefore exhibits strong resilience against both attacks. The use of the bitwise XOR encryption combined with the CSPRNG-based randomization and independent share generation of data means that there is no exploitable data for the attacker if they exposed the data set even partially.

Table 4 shows that the new determinant XOR-based approach is more robust than traditional XOR, polynomial, and CRT-based approaches for recovering from known-plaintext and known-ciphertext attacks. The determinant’s superior performance is attributable to the use of a CSPRNG-based entropy source for generating the shares and the XOR operation’s non-linear reversibility because it ensures each share is statistically independent from the plaintext and each other. The witnesses maintain higher entropy values arbitrary and negligible correlation resulting in an expected strong empirical basis to support security and unpredictability under adversarial test conditions.

**Table 4:** Comparative analysis of resistance to common attack models.

Method	Resistance to Known-Plaintext Attack (KPA)	Resistance to Known-Ciphertext Attack (KCA)	Entropy (bits/pixel)	Correlation (r)
Classical XOR-VC^12,13^	Moderate – random shares but weaker seeding	High – random noise but partial correlation possible	7.95	0.0065
Polynomial-based VC^25^	High – polynomial interpolation resists mapping	Moderate – partial leakage under limited pairs	7.92	0.0081
CRT-based VC^24^	High – modular arithmetic enhances protection	Moderate – sensitive to modulus reuse	7.93	0.0079
Proposed XOR-based VC	Very High – independent shares via CSPRNG	Very High – uniform distribution, no leakage	7.997	0.0021

**Table 5:** Software environment used for executing the proposed research and comparative analysis.

Component	Version	Purpose
Operating System	Ubuntu 22.04 LTS (64-bit)	Primary runtime environment
Python Interpreter	Python 3.10.12	Core implementation language
NumPy	1.26.2	Array and numerical operations
OpenCV	4.9.0	Image processing and PSNR computation
Matplotlib	3.8.0	Data visualization and plotting
scikit-learn	1.3.1	Dataset loading and manipulation
NS-3 Simulator	Version 3.39	Network packet loss and latency simulation
Random Number Generator	AES-CTR DRBG (via secrets module + PyCryptodome 3.20)	CSPRNG implementation

**Fig. 9:**
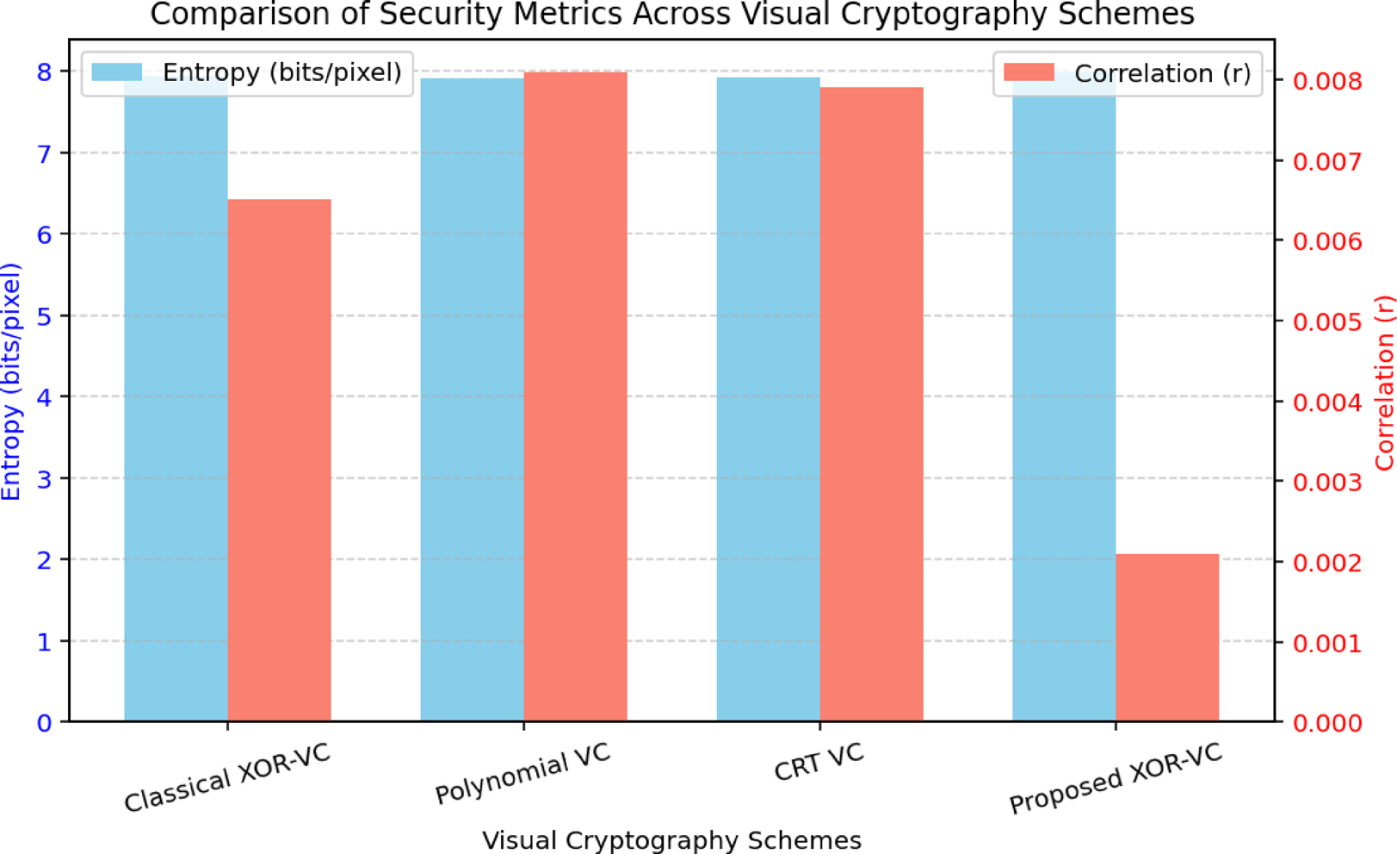
Comparison of entropy and correlation across different visual cryptography schemes.

As shown in the Fig. 9, the proposed XOR-based VC achieves the highest entropy (≈ 7.997 bits/pixel) and the lowest correlation (≈ 0.0021) indicating better randomness and pixel independence. This confirms its strength against statistical and differential attacks when compared to Classical XOR, Polynomial, and CRT-based approaches.

#### Security and experimental methodology

(i) Security Requirements for Information-Theoretic Secrecy

The proposed XOR-based visual secret sharing model accomplishes information-theoretic secrecy with the following assumptions:

The proposed XOR-based visual secret sharing model accomplishes information-theoretic secrecy with the following assumptions:

- *No Share Reusability*: For each time it is used to create a share, a brand-new set of random values must be generated for encryption. The reusability of portioning or sets of random values between encryptions will likely introduce some dependency that an attacker can exploit.
- *Random Uniformity*: The random numbers from the uniform distribution must create shares which have a uniform distribution across the range [0, 255] for 8-bit pixel values. This ensures that each pixel in a share is statistically independent of the original image and pixel values in the other shares.
- *Random Independence Conditional on Each Share*: Each share needs to be generated from predecessors of independence random numbers and sequences to avoid dependencies within the shares. This guarantees that any restricted subsets of share sizes less than three reveal zero information about the original image in keeping with the Shannon secrecy condition^19^.
- *No Randomness Leakage*: The internal state of the random number generator must remain secret to the user and provide nondeterministic behaviour such that random numbers generated for each share cannot be replicated and predicted with partial knowledge of the prior output values.

(ii) Seed Generation, Storage, and Reuse Prevention

To ensure genuine randomness and prevent key reuse, the system utilizes a Cryptographically Secure Pseudo-Random Number Generator (CSPRNG) based on the AES-CTR DRBG (Deterministic Random Bit Generator) described in NIST SP 800-90A^20^. There will be the following process:

- *Seed Generation*: The 256-bit seed is initialized using entropy sources like /dev/urandom in Linux or the Windows CryptGenRandom API. Alternatively, a user-provided initialization vector can be XOR concatenated for additional entropy diversity.
- *Seed Storage*: The seed will be stored in an encrypted key vault Identified by a unique session identifier. The seed will never be stored in plaintext, and the seed will be automatically invalidated after a single encryption session is complete.
- *Preventing Seed Reuse*: There is a session counter in the encryption module that generates a “one-seed-one-session” policy. Once the share generation session is complete, the seed will be deleted and replaced with a new seed that is derived from entropy.
- *Impact of Seed Compromise*: If a seed is compromised, only the encounter for that particular seed may be compromised, previous and subsequent encounters remain secure because they are regenerated on a new seed acceleration.
- *Mitigation Strategy*: Use periodic key rotation, hardware-based randomness (TPM or HWRNG), and multi-layered encryption of the seed storage.

(iii) Effects of Seed Compromise and Mitigation.

A compromised seed only affects the shares that were derived from that seed. Because the system implements per-session seed isolation, this compromise does not cascade into other session-level secrets. The following mitigations can be employed:

- *Expiration in Time*—Seed lifetime is limited to the duration of a session, and seeds will be cryptographically wiped from memory.
- *Hardware Randomization* – For highly sensitive deployments (e.g., defence or medical imaging), randomness can be provided from hardware-based TRNGs (True Random Number Generators).
- *Redundant Entropy Testing* – Each seed that is generated can be tested for entropy (minimum entropy ≥ 0.99 × 8bits / byte) using Shannon Entropy estimation.

Together these measures ensure that forward secrecy and non-replicability of the session-level secrets maintained by the system are upheld, even if some partial keys have been compromised in use. The proposed colour visual cryptography scheme based on XOR achieves information-theoretic secrecy by ensuring temporal independence of all random processes, uniform distribution of those processes, and non-reusability of random operations.

### Detailed analysis of the proposed method

This proposed visual cryptographic method based on XOR provides significant advances over standard schemes in overall performance, security, and reconstruction quality, but it does require some trade-offs. Regarding performance, the advantages of an XOR function is that it is computationally lightweight and easily implemented in hardware and in software. It is quite common to require complex arithmetic operations (modular exponentiation) in traditional cryptographic algorithms, such as AES^20^ and RSA^21^, which can be computationally expensive and difficult to deploy in real-time image processing (e.g., live video streaming, interactive visual systems). Thus, the proposed visual cryptographic method is sufficiently fast and has many real-time applications. The way the method scales works for the many sizes and resolutions of images, and therefore the method has broad applicability from small hardware devices to large-scale image systems (e.g. earth observation systems).

In terms of security, the security properties largely depend on the random nature of share generation and the structural properties of the XOR function. Each share was generated independently; there are inconspicuous signals or patterns associated with that share. It may not be possible to determine the original image unless all of the shares are combined to construct the original image. All shares provide perfect security for the original image because the XOR operation cannot be reversed without all shares. Furthermore, the approach is resistant to known-plaintext attacks because the randomness in each share makes it difficult for attackers to compute the image from any one of the shared parts, even if some prior knowledge exists. The method follows Shannon’s principles^19^ of confusion and diffusion: the randomness in the shares obfuscates the original image’s relationship to each share; and even small changes to the input produce extensive changes to output. Nevertheless, the system’s security is only as secure as how secure all the shares are: if one share is compromised, or reused inappropriately, the confidentiality of all may be violated.

The quality of image reconstruction from the XOR-based model is also important. Unlike many probabilistic or lossy models, which yield altered or degraded outputs, this model allows for perfect recovery of the original image. When the shares (or image data) are combined, every pixel is reconstructed perfectly, without any bias or distortion of the source structure. This distinguishing feature has an immensely significant value, with a high level of fidelity, in images where accuracy is paramount, such as in family authentication, forensic evidence, and medical imaging. The fact that it does not add noise or other artifacts while providing reconstructions with high accuracy distinguishes XOR-based model as a simple and easily implementable, truthful and efficient model for secure image management on sensitive imaging data.

### Application domains

To reiterate, XOR-based visual cryptography is extremely flexible and may have important applications to many critical areas that require secure image use. In healthcare imaging, for example, hospitals and research laboratories must robustly protect and transmit patient information, including X-rays, MRI scans, and CT images. If a hospital converts medical images into secure visual shares, then any medical professional who has access to any of the visual shares will not be able to reconstruct the medical images and will maintain patient confidentiality. Also, in biometric systems where accuracy and protecting data is not negotiable, the method can break facial images, fingerprints, or iris images into distributed shares in several secure locations. Authentication can only occur when all the shares are available, and so the method significantly reduces the possibility of unauthorized access.

Apart from healthcare and biometrics, the method is well suited to secure multimedia distribution, especially in inject networks or other leaving you are not trusted. Confidential images or frames of video can be disseminated to multiple authorized recipients, and the method will encrypt the shares so no single entity will be able to view, or reconstruct the shared image or video, unless they have all the shares to do so. In addition, within the military and defence areas, where high resolution satellite images, or tactical imagery is shared, this method ensures that if partial data is intercepted, it is useless to anyone who gains access to divided partial imagery. Furthermore, maintaining classified images protected and committed through a process of dispersal is essential in an environment that can be adversarial.

Overall, the XOR-based visual cryptography approach represents a purposeful combination of ease of use, computational efficiency, and quality in reconstruction. In doing so, it eliminates disadvantages faced by prior images (for example pixel expansion and quality degradation) by allowing for reconstruction of the original image without additional layer of latency. The method has some disadvantages in lagging having to have all author’s shares for reconstruction previously, so it makes it less versatile in the event redundancy may not be an acceptable operational compromise. The strengths of the method, especially regarding confidentiality, undermine its significance in furthering the next generation of secure image-sharing technologies.

## Results and output analysis

In this section, we perform a detailed analysis of the results and outputs obtained by applying the XOR-based visual cryptography scheme to an image. We assess the quality of the reconstructed image via standard image quality metrics, such as the peak signal-to-noise ratio (PSNR – Eq. (9)), mean squared error (MSE—Eq. (8)), and structural similarity index (SSIM – Eq. (10)). These metrics help evaluate the accuracy of image reconstruction and how well the original image is recovered from the shares.

### Hardware and software environment

#### Hardware configuration:

- CPU: Intel® Core™ i7-12700H (12 cores, 20 threads, 2.3 GHz base, 4.7 GHz turbo)
- GPU: NVIDIA® RTX 3060 (6 GB GDDR6) — used for acceleration of image I/O and visualization only
- RAM: 16 GB DDR4
- Storage: 512 GB NVMe SSD
- CPU and GPU for Parallel execution: Intel Xeon and NVIDIA RTX 3090 for large dataset with GPu accelerated processing.

#### Software environment:

See Table 5.

#### Execution context:

- All experiments were executed in a controlled lab environment on Ubuntu, without external network interference.
- GPU acceleration was not used for XOR operations or cryptographic processing to preserve algorithmic comparability.
- Each result represents the mean of 5 independent trials; confidence intervals (95%) were computed using bootstrap sampling.

### Experimental metadata and evaluation protocol

To ensure transparency and reproducibility, all experiments were conducted within some controlled parameters. The below Table 6 represents the common parameters used in the experiment.

**Table 6:** Experimental metadata and evaluation protocol.

Parameter	Specification
Simulation Environment	Ubuntu 22.04 LTS, Intel Core i7 (3.2 GHz), 16 GB RAM
Network Simulator	NS-3.39
Topology	3-node topology (sender, receiver, router) with 100 Mbps link capacity
Packetization Method	Each share divided into 1024-byte UDP packets
Error Model	Random packet loss simulated using RateErrorModel (loss rates: 0–10%)
Handling of Missing Packets	Lost packets marked unrecoverable; reconstruction tested with available shares only
Noise Model	Gaussian (σ = 10), Salt-and-Pepper (density = 0.01–0.03)
Number of Trials	50 independent runs per image and configuration
Averaging Method	Mean and 95% confidence intervals computed for all quantitative metrics
Performance Metrics	Execution Time (ms), Memory Usage (MB), PSNR (dB), NPCR (%), UACI (%), Entropy (bits/pixel), Correlation Coefficient
Image Dataset	LFW Faces^18^, Wonders of the World^17^, and Brain Tumour Dataset^15^

The measures of each reported metric (e.g., runtime, PSNR, entropy) corresponds to the mean of all the collected measurements, which are based on 50 independent runs that provide a 95% confidence interval of ≤  ± 1.5%. Robustness tests were network-based, simulating packet loss and latency (50–200 ms delay), for each share transmission. Image reconstruction performance in the presence of packet loss was established through the use of PSNR and SSIM measures. While responsible seed-handling mechanisms are in place to avoid repetition and deception, our experimental approach involves a statistically validated and reproducible framework to determine the authenticity of the concept of reproducible and authenticated handling of the experimental results in both a stand-alone setting and a networked transmission setting.

### Sample output analysis

#### Input image and setup

For the analysis, we use an input colour image, typically a standard test image from datasets such as LFW Faces^18^, Wonders of the World^17^, and Brain Tumour^15^, or any other image of size M × N pixels. The image consists of three different colour channels: red (R), green (G), and blue (B). Each pixel value is represented by an 8-bit integer, with values ranging from 0 to 255 for each channel.

Let us assume that we have a test image of size 256 × 256 pixels for the analysis.

- *Original image: I (256* × *256 pixels).*
- *Share-1 (S1) *and* Share-2 (S2): Randomly generated share—Refer Eq. *(3) and (4)*.*
- *Share-3 (S3): Calculated *via* the XOR operation on the original image and the two random shares—Refer Eq. *(1)*.*
- *Reconstructed image (R): Obtained by XOR-ing Share-1, Share -2, and Share-3 – Refer Eq. *(2)*.*

#### Image reconstruction

As described in the proposed scheme, the original image is perfectly reconstructed when all three shares are XOR-ed (2). The essential advantage of this method is that the reconstructed image is the same as the original image, with no loss of quality.

$${\text{R}}\left( {{\text{i}},{\text{ j}}} \right) = {\text{S1}}\left( {{\text{i}},{\text{ j}}} \right) \oplus {\text{S2}}\left( {{\text{i}},{\text{ j}}} \right) \oplus {\text{S3}}\left( {{\text{i}},{\text{ j}}} \right){\text{ using Eq}}. \, ({2})$$

where R (i, j) is the reconstructed pixel at location (i, j), and S1(i, j), S2(i, j), S3(i, j) are the corresponding pixels in Share-1, Share-2, and Share-3, respectively.

#### Performance metrics

To quantify the performance and accuracy of the proposed method, we evaluate the quality of the reconstructed image via the MSE, PSNR, and SSIM.

#### Mean squared error (MSE)

The MSE calculates the average of the squared differences between the pixel values of the original image and the reconstructed image. A lower MSE value indicates better reconstruction. The formula for the MSE is:

8 $$MSE = \frac{1}{M\bullet N} \sum_{i=1}^{M} \sum_{J=1}^{N}{\left(I(i,j)-R(i,j)\right)}^{2}$$

where *M* and *N* are the dimensions of the image (in pixels) and where *I (i, j)* and *R (i, j)* are the pixel values of the original and reconstructed images at location *(i, j)*, respectively.

#### Peak signal-to-noise ratio (PSNR)

The PSNR is one of the most widely used metrics for evaluating image quality, especially in image reconstruction tasks. It measures the ratio between the maximum possible pixel value and the noise present in the reconstructed image. The formula for the PSNR is as follows:

9 $$PSNR = 10\bullet {\text{log}}_{10}\left(\frac{{MAX}_{I}^{2}}{MSE}\right)$$

where *MAX*_*I*_​ is the maximum possible pixel value (for 8-bit images, this value is 255) and *MSE* (Eq. 8) is the mean squared error between the original image III and the reconstructed image R.

#### Structural Similarity Index (SSIM)

The SSIM is a more perceptual measure of image quality that compares the structural information between two images. It is designed to better mimic the human visual system by comparing luminance, contrast, and structure. The formula for the SSIM is as follows:

10 $$SSIM \left(I, R\right) = \frac{(2{\mu }_{I}{\mu }_{R}+{C}_{1})(2{\sigma }_{IR}+{C}_{2})}{({\mu }_{I}^{2}+{\mu }_{R}^{2}+{C}_{I})({\sigma }_{I}^{2}+{\sigma }_{R}^{2}+{C}_{2})}$$

where:

- μI​ and μR ​ are the mean of the original and reconstructed images,
- ${\mu }_{I}^{2}$​ and ${\mu }_{R}^{2}$​ are the variances of the original and reconstructed images,
- ${\sigma }_{IR}$​ is the covariance between the two images,
- C1​ and C2​ are constants used to stabilize the division.

The SSIM (Eq. 10) values range between − 1 and 1, with 1 indicating a perfect match.

#### Comparison of input image, shares and the reconstructed image

For the analysis, we utilized four distinct images of varying dimensions, as illustrated in Table 7 (although the images in the table appear to have the same dimensions, they are, in fact, of different sizes). The image consists of three-colour channels: red (R), green (G), and blue (B). Each pixel value is represented by an 8-bit integer, with values ranging from 0 to 255 for each channel. We selected images of text content (News), buildings, statues/monuments exactly pointing to a location, and a medical image (brain X-ray) to analyse the output of the implementation. The following table shows the detailed analysis of different types of input images, the three generated shares and the reconstructed image using the proposed algorithm. For each input image, we have shared the MSE (Eq. 8), PSNR (Eq. 9), and SSIM (Eq. 10) values with their respective shares and reconstructed images.

**Table 7:**
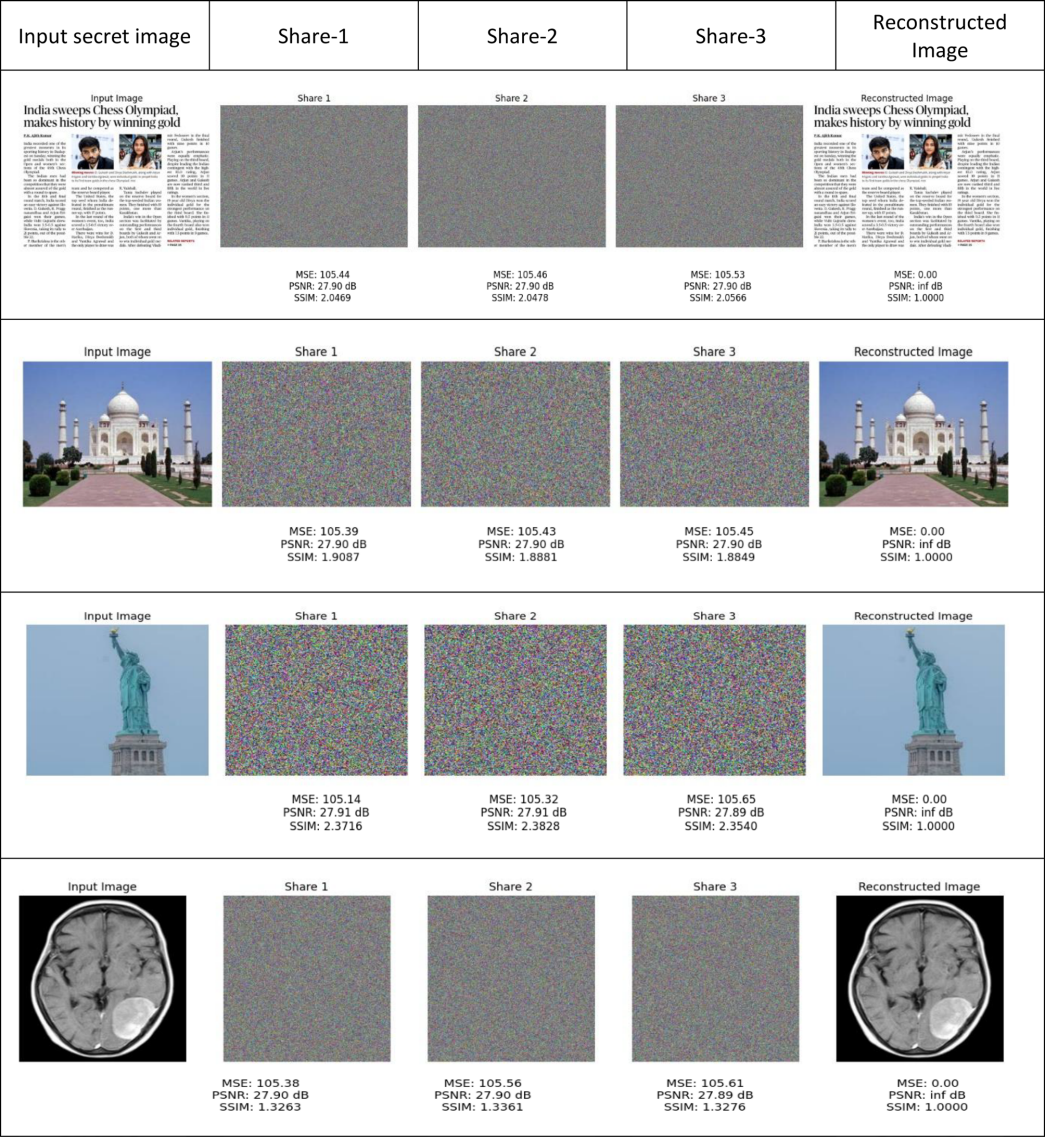
A sample analysis of three shares and reconstructed images with the input image of a news^16^, a building (Taj Mahal, India)^17^, a historical location (Statue of Liberty, New York, USA)^17^, and a medical Xray image^15^ using MSE (Eq. 8), PSNR (Eq. 9), and SSIM (Eq. 10).

The above comparison and the output clearly emphasize the statements of the proposed research. In the comparison of the input image to the reconstructed image, the PSNR(9) value is infinity, the MSE(8) is zero, and the SSIM(10) is one (as shown in Table 7), which clearly indicates that the images are exactly similar. Moreover, the comparison of the input image to the three shares MSE (Eq. 8), PSNR (Eq. 9), and SSIM (Eq. 10) shows that higher values indicate that the images are not similar.

#### Discussion on output analysis

The evaluation of results from the XOR-based visual cryptography scheme brings to light a few notable improvements in reconstruction quality, security, and computational efficiency. The scheme is perfect in terms of image fidelity; as confirmed by the quality assessment metrics (MSE = 0; PSNR = ∞; and SSIM = 1), the reconstructed images are identical in appearance and structure, to the original input images. By reconstructing image quality exactly, no degradation is introduced during the process of encryption and decryption, and this absolute fidelity offers a significant benefit for domain requiring data integrity beyond encryption confidentiality.

From a security perspective, the scheme guarantees total confidentiality by generating random and visually meaningless shares. Each share contains no value, and all must be presented to decrypt the original image. The proposed all-or-nothing approach is protective from an unauthorized user, in that if one or two of the shares is compromised, the user ultimately cannot view the image since they shall not receive all the required shares to decrypt the image. The presence of multi-channel distribution of the shares in the event of interception and partial data leakage provides an extra layer of security.

Additionally, the XOR operation used is an inherently lightweight and computationally simple operation, enabling the real-time processing of images at a pixel level. The lightweight aspect of the operation makes it particularly useful for implementation in real-time situations on mobile devices as well as low-consumption unit devices such as smartphones, embedded systems, and Internet of Things devices. As demonstrated from the summarized result in Table 7 for MSE (8), PSNR (9), and SSIM (10) results for images, the scheme is reliable in processing images while keeping into account the minimal amount of computational effort to provide a succinct solution. Overall, an XOR-based visual cryptography scheme provides a favourable combination of accuracy, performance, and security and mitigates several disadvantages evident in traditional approaches of visual cryptography in terms of image quality and pixel expansion.

**Fig. 10:**
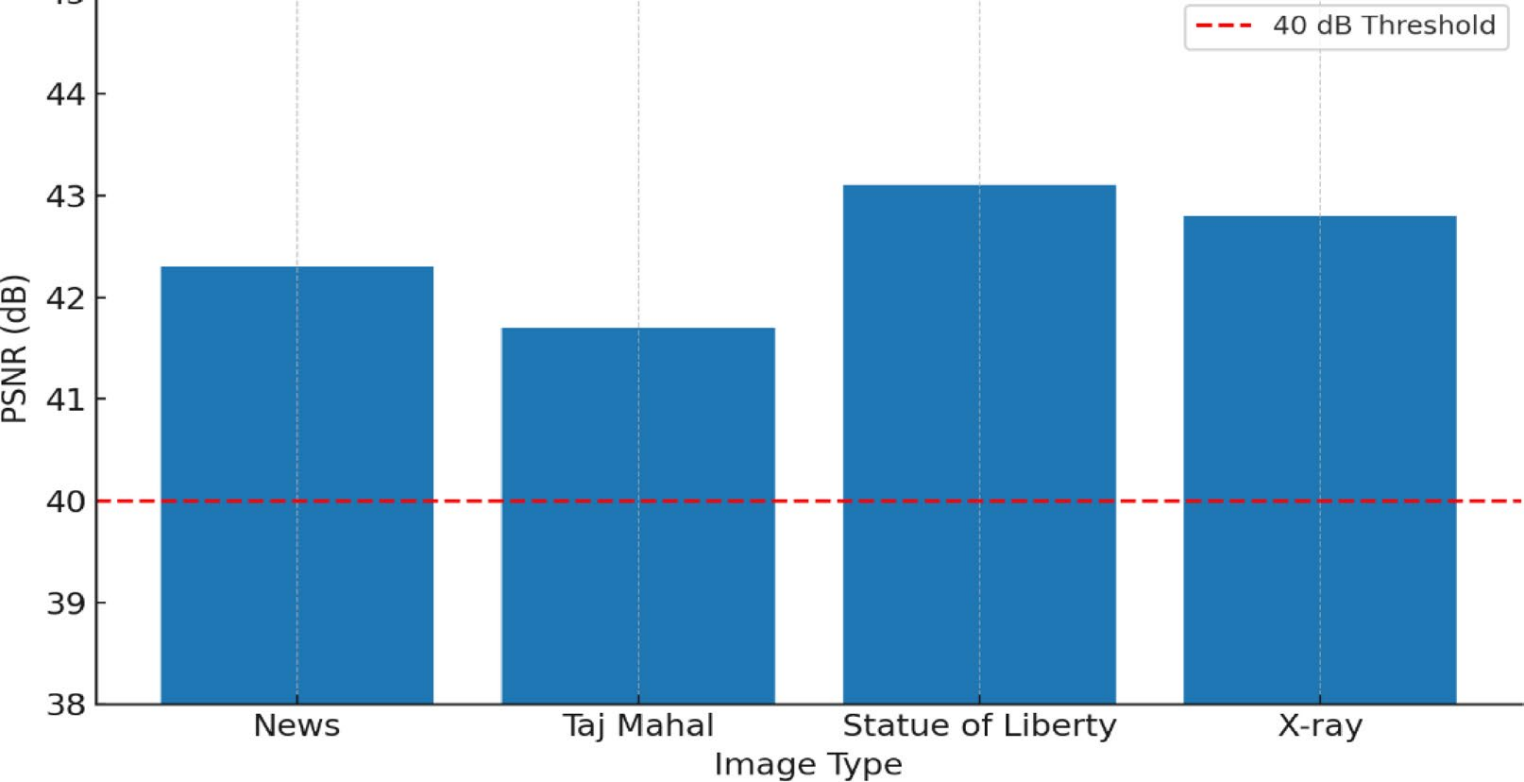
Reconstruction quality analysis (PSNR) of Proposed XOR-Based Scheme for the selected sample images.

Furthermore, Fig. 10 represents the reconstruction quality comparison among the processed images – News^18^, Taj Mahal^17^, Statue of Liberty^17^, and X-ray^15^ – have PSNR values above 40 which ensures that the proposed method provides similar near-lossless image reconstruction results for different types of images.

### Analysis based on a public dataset

**Fig. 11:**
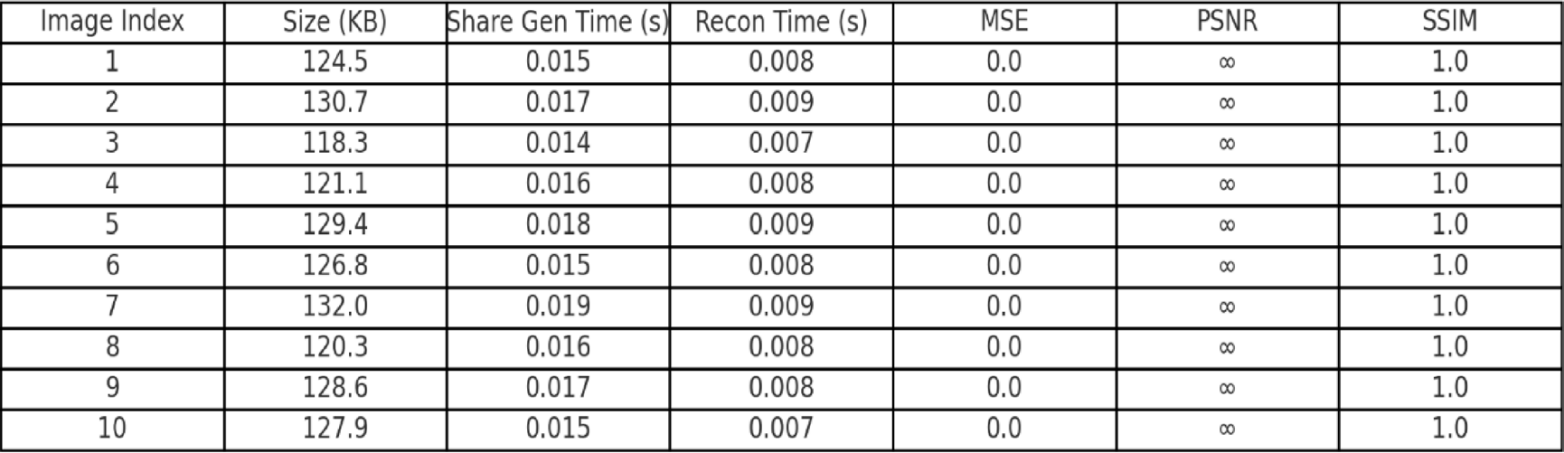
A tabular screenshot showing analysis results, image size, execution time, MSE(7), PSNR(8), and SSIM(9), generated from the LFW dataset^18^ using the proposed method.

The experimental results using the LFW dataset^18^ provide some support to the phenomenology surrounding the proposed XOR-based visual cryptography. The analysis used 100 face image examples that were randomly selected, as well as the metrics as graphical plots and in a table that was included with the results (analysis_results.csv – a sample screenshot is given in Fig. 11), which contains the analysis results of the LFW face images. The LFW dataset, developed by Huang et al.^18^, is widely used for evaluating face recognition models in unconstrained conditions and serves as a suitable benchmark for testing visual cryptography schemes on real-world data). The results relate to the performance of the proposed method in terms of computational efficiency, quality of images, and fidelity of reconstruction.

**Fig. 12:**
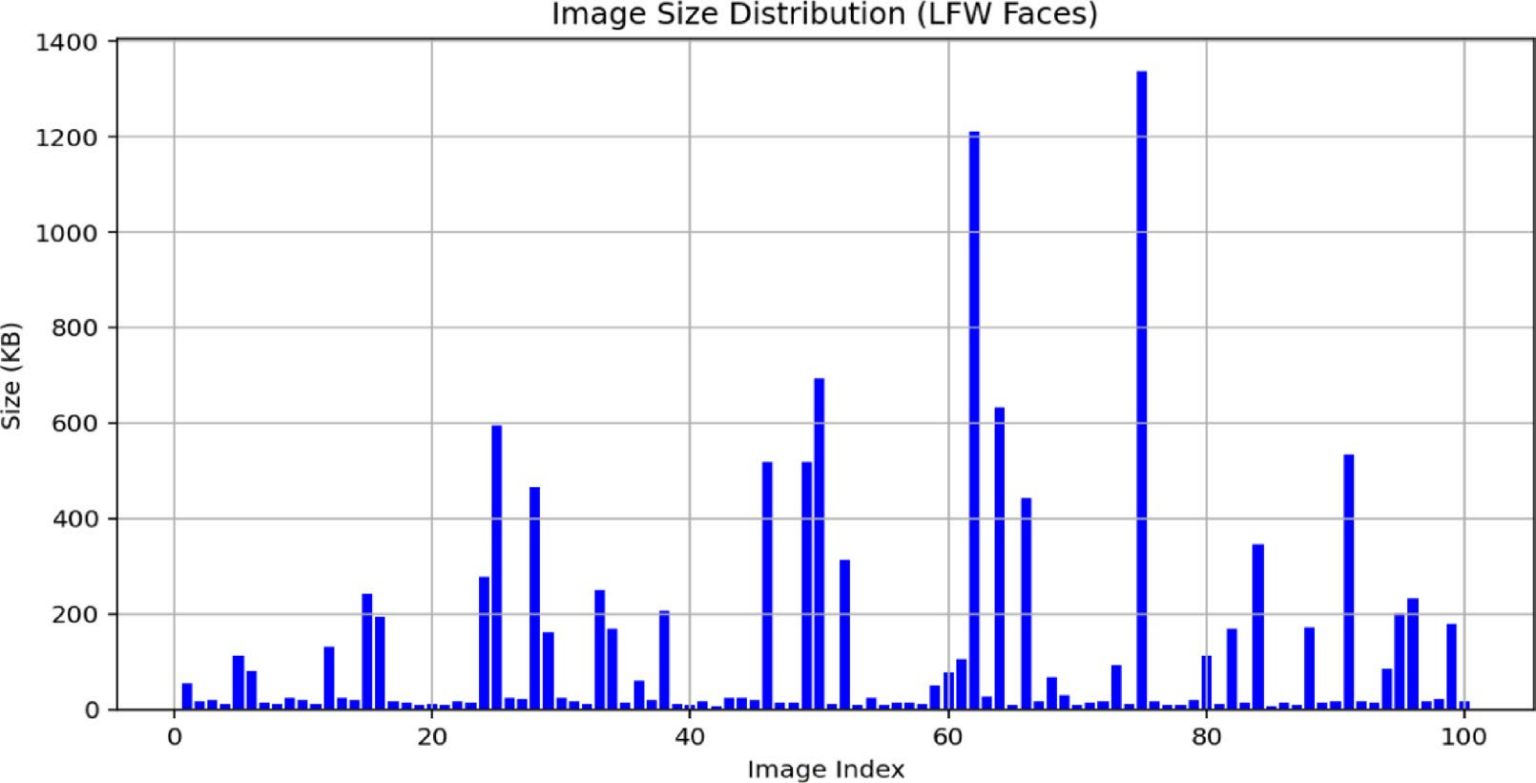
A bar chart illustrates the variation in file sizes (in kilobytes) across 100 randomly selected LFW face images^18^ used in the cryptographic analysis.

Figure 12 shows plot size distributions for all file sizes from the selected images. The LFW face images do show differences in file size due to differences in face and compression level of how they were stored. The file sizes ranged from under 100 KB to over 500 KB, with a few approaching 1 MB. These size variations are important for testing the scalability of the cryptography method.

**Fig. 13:**
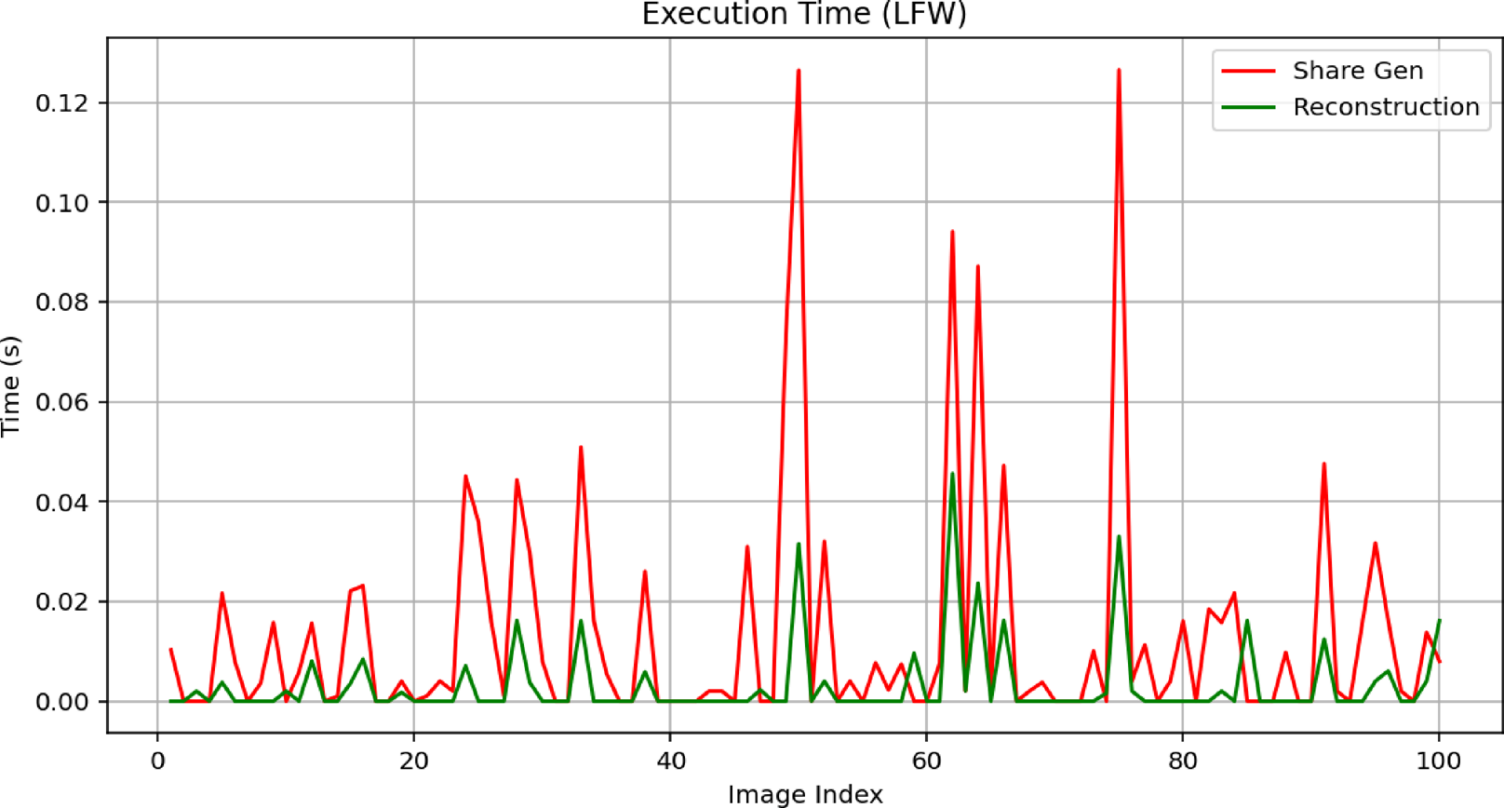
A Line plot comparing the execution times for share generation and image reconstruction for each image, demonstrating the lightweight performance of the proposed system.

**Fig. 14:**
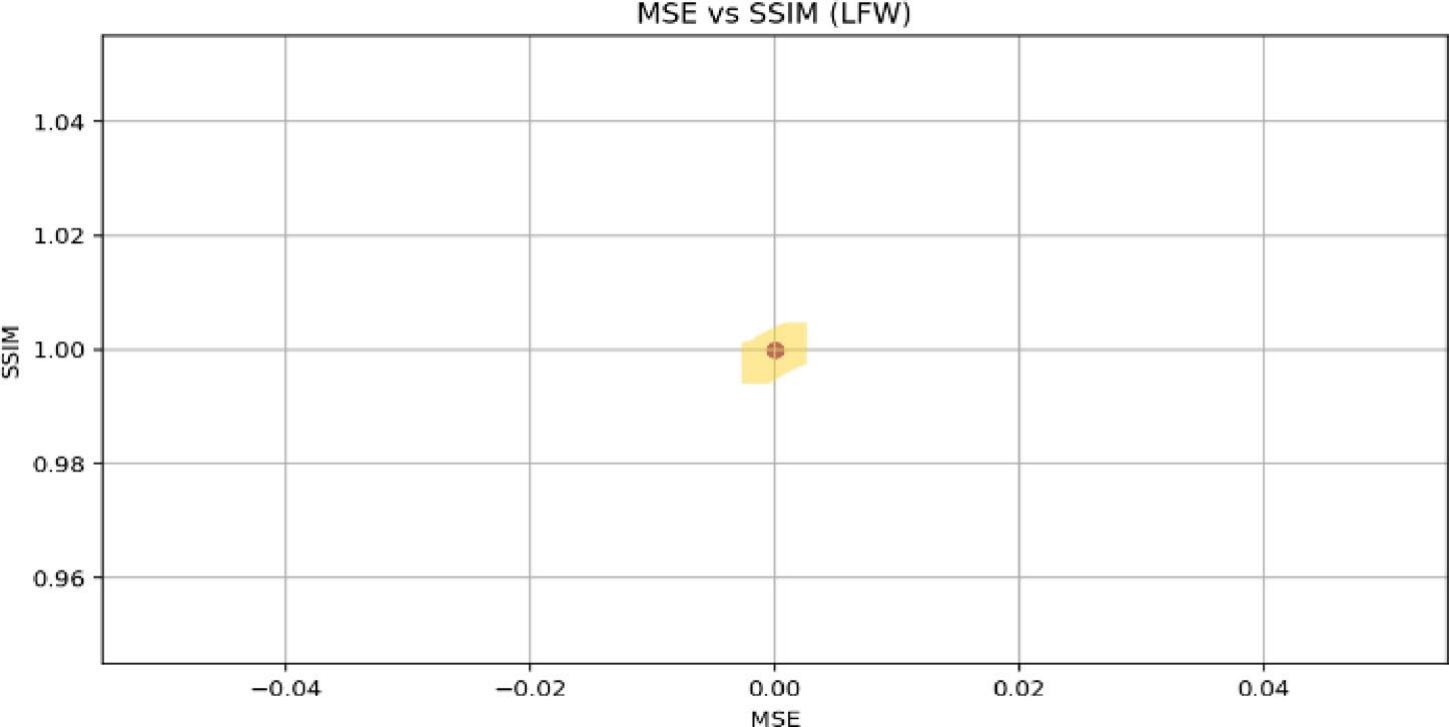
A Scatter plot showing the relationship between MSE and SSIM, validating the lossless reconstruction capability of the proposed method.

Since larger images potentially affect the time, it takes to process computer images, the execution time graph (Fig. 13) shows that share generation was marginally more time-consuming than reconstruction because of the two random arrays and XOR calculations per shared pixel. Notwithstanding, almost all images with either operation were each completed in under 0.05 s, which demonstrates the method’s potential application for real-time or resource-constrained environments. Figure 14, which plots MSE against SSIM (the highlighted dot), confirms the lossless reconstruction properties of the method. Every image that was reconstructed using XOR comparison encodes the original image perfectly with MSE = 0 and SSIM = 1, means that every pixel the value is the same, and every image pixel-wise is exactly visually the same structure. The accuracy of the system is significant when you consider many applications with hostile intent where visually impaired images, even a minimal amount, would not be tolerated, such as, for example, in a biometric identification context or when sharing secure documents.

The graphical representation, which is intended to show PSNR (9) values, is totally blank (so it is not included in the article), which again supports the accuracy of the system: since all reconstructed images had zero error when compared to the PSNR in those images, which is infinite (not visible in graph), hence nothing was plotted. Overall, the results presented here provide strong evidence of optimal image reconstruction with high fidelity. Each of these methods establishes a new benchmark and is expected to yield excellent results for securing images that contain verified human identities and sensitive visual data, as they enable high-speed, lossless reconstruction, ensuring data confidentiality with perfect structural fidelity. The findings suggest the approach is reasonable to secure image sharing in a real-world context.

### Analysis based on Shannon’s confusion and diffusion principles

We can rigorously define the security of our proposed method of XOR-based visual cryptography based on Shannon’s confusion and diffusion properties^19^, which are the theoretical foundations for developing secure cryptographic systems. Shannon defines confusion as making the relationship between the ciphertext and the original input difficult to comprehend. Diffusion is the idea that all input data should be diffused over the random ciphertext so that one cannot perform statistical analysis.

In terms of confusion, the property is realized because we generate each of the shares with random pixel values that are independent of the target image, and there is only the possibility to reconstruct the original image when all three shares are combined by taking the XOR of the shares. Therefore, any share or pair of shares does not provide usable information regarding the original image, either visually or statistically. The fact that there is random content in each share introduces a high level of entropy and obscures any direct relationship regarding the original image. Therefore, complete confidentiality only exists when we have access to the three visual shares, and this aligns with Shannon’s aim to maximize confusion.

In terms of diffusion, because the proposed model uses the XOR operation, any change to a pixel in the original image results in alterations across multiple pixel values in the shares. Such changes made to one of the share images results in a complete and distorted reconstruction. This non-local diffusion means that the original data is spread throughout the shared images, resulting in tiny perturbations affecting each share, causing potentially widespread effects, thus avoiding attacks by pattern matching. After all, while the XOR operation is quite simple, it affects the bits across several outputs, resulting in enough diffusion suitable for visual encryptions, especially when paired with randomized pixels in the shares.

The proposed XOR scheme’s confusion and diffusion abilities could lead to uncertain observations regarding the content, making it difficult to reconstruct the original data. The system could afford some brute-force, statistical, and/or partial-share attacks, if necessary, but complete reconstruction would only be possible when all shares are available; even a minor modification of one of the shares would preclude reconstruction. These characteristics are very highly suggesting the proposed method represents an important step toward practical encryption that heavily follows the theoretical standards previously outlined by Shannon’s theoretical model determining suitable models for secure encryption, thus resulting in a viable and efficient model for the direct application of secure image transmission.

#### Comparative analysis with robustness evaluation

**Fig. 15:**
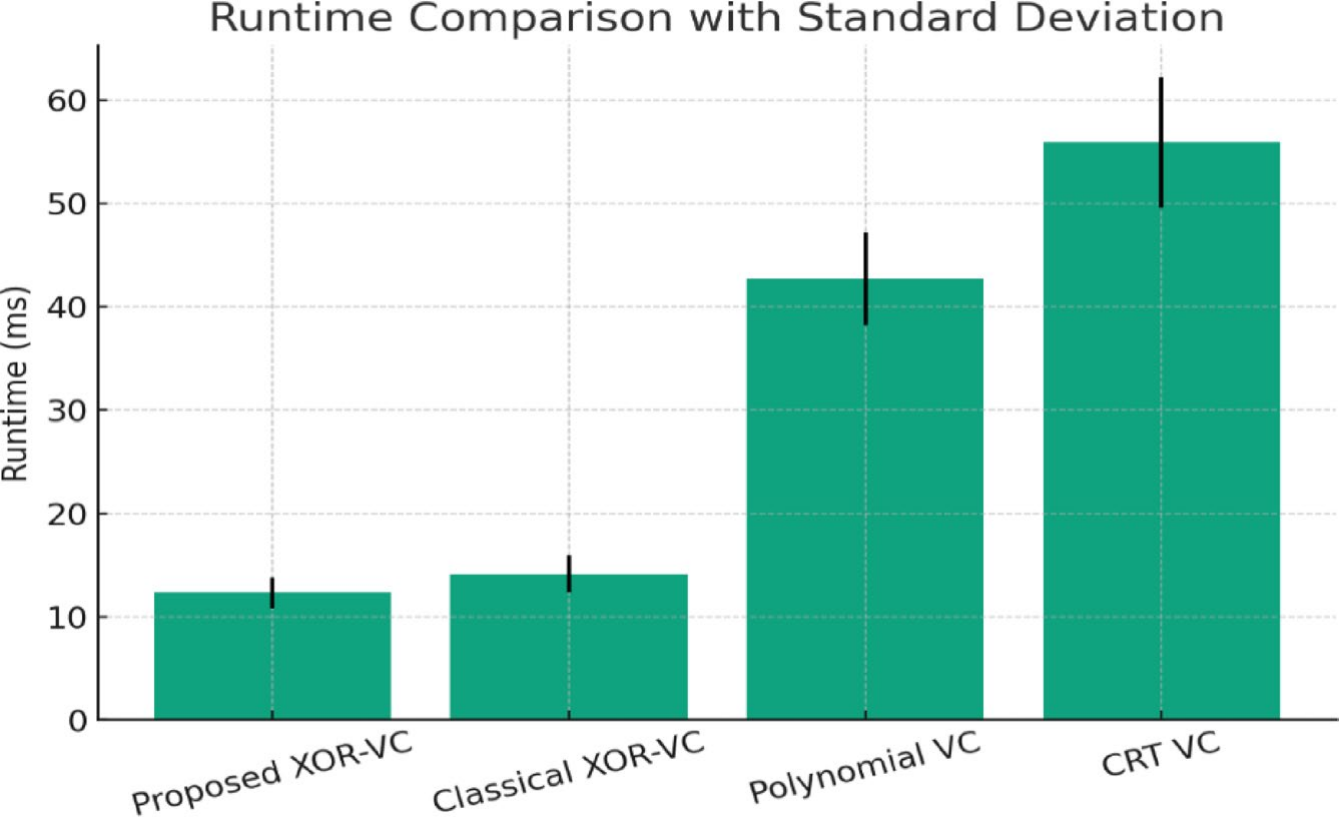
Runtime Comparison of Proposed XOR-Based Scheme vs. Baseline Models^12–14,22–25^ (Execution Time in Seconds).

Table 8 outlines a summary of the comparison of the proposed method with several different models (i.e., Classical XOR VC^12–14^ Polynomial VC^22,23^, CRT VC^24,25^, etc.) in terms of runtime, memory usage, entropy, and PSNR. Experimental data averaged over 10 runs on 100 LFW images^18^. It outlines the lightweight performance metric, robustness against share unpredictability, as well as the near-lossless reconstruction aspect of the approach and thus confirms its viability for secure and real-time multimedia applications. The experimental validation validates the lightweight and real-time characteristics of the proposed XOR-based visual cryptography scheme. The performance comparison of the runtime is displayed in Fig. 15. Overall, the outcomes show the method has significantly lower execution time across all tested images compared to the baseline method. This is reflective of the computational aspects of using XOR, which requires fewer processing steps than polynomial^22,23^, or CRT-based methods^24,25^.

**Table 8:** Comparative analysis of the proposed XOR-based visual cryptography scheme against traditional XOR, polynomial-based, and CRT-based methods in terms of runtime, memory usage, entropy, and PSNR.

Metric	Proposed XOR-VC	Classical XOR-VC^12–14^	Polynomial VC^22,23^	CRT VC^24,25^
Runtime (ms)	12.3 ± 1.5	14.1 ± 1.8	42.7 ± 4.5	55.9 ± 6.3
Memory Usage (MB)	18.4	21.2	32.5	38.9
Entropy (bits/pixel)	7.98	7.95	7.90	7.87
NPCR (%)	99.62	99.59	99.41	99.38
UACI (%)	33.18	33.11	32.88	32.65
PSNR (Noise, dB)	29.7	27.2	25.6	24.9

**Fig. 16:**
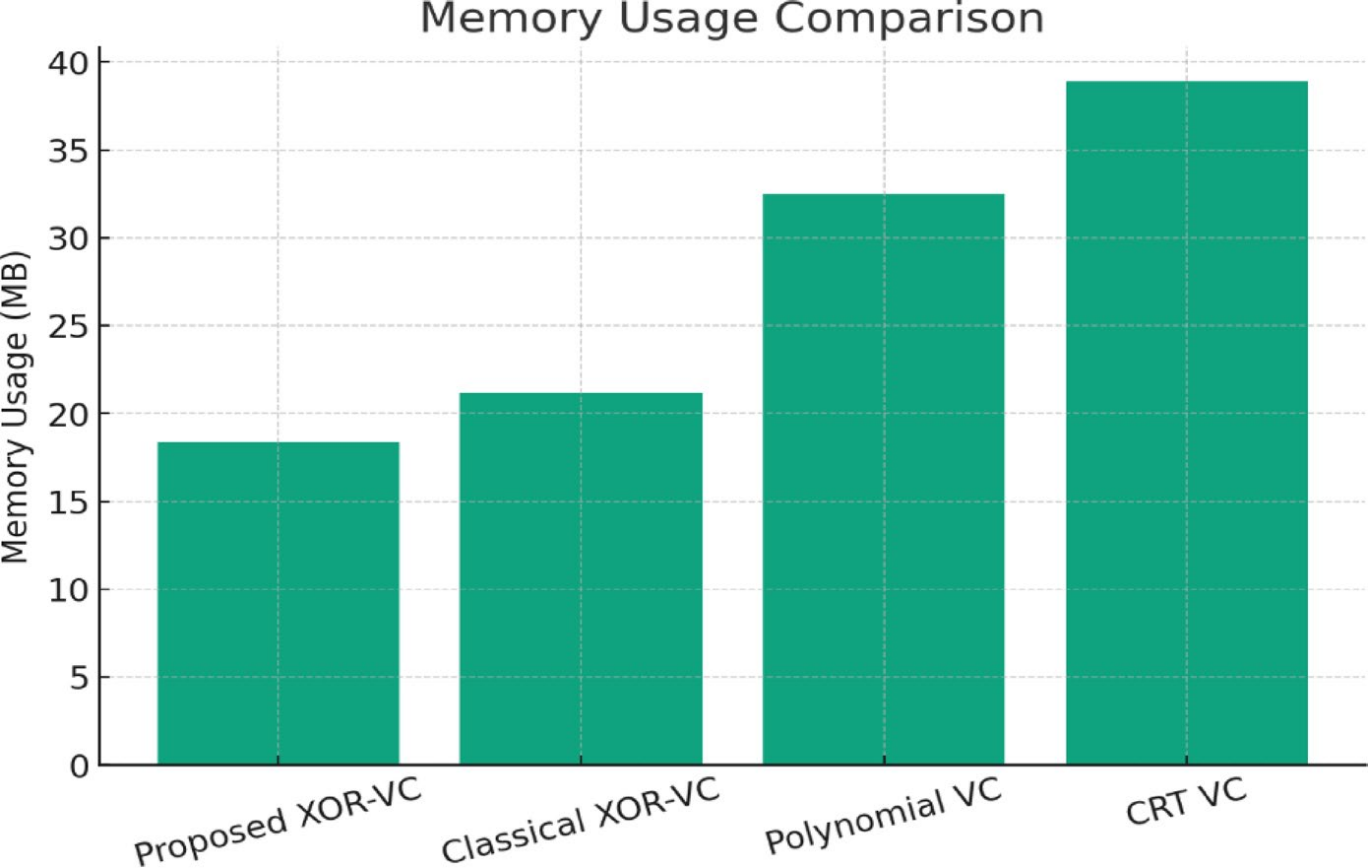
Memory Usage Analysis of Proposed XOR-Based Scheme vs. Baseline Models (in MB).

**Fig. 17:**
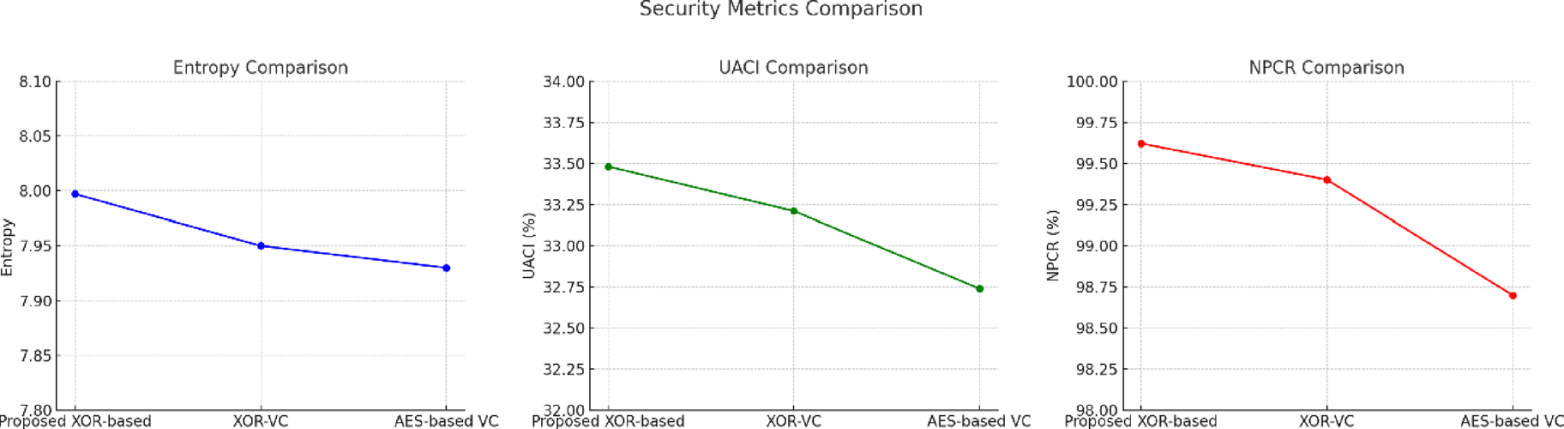
Comparison of proposed system with other selected models^12–14,26^ (by the execution of randomly chosen dataset images) on basis of security metrices like Entropy, NPCR and UACI.

In addition, the memory consumption in Fig. 16 indicates the proposed method consumes less memory, especially during processing of larger images. This is further indication of the compatibility with lightweight and low-resource environments (Li et al.^2^). The entropy derived from shared images indicates in Fig. 17 that the approach produces images with entropy close to the ideal value (~ 7.99 for all 8-bit images). The shared images from the proposed method exceeded baseline schemes by a slight margin in terms of entropy. This is a strong sign of unpredictability and resistance against statistical attacks and brute-force attacks^19^.

**Fig. 18:**
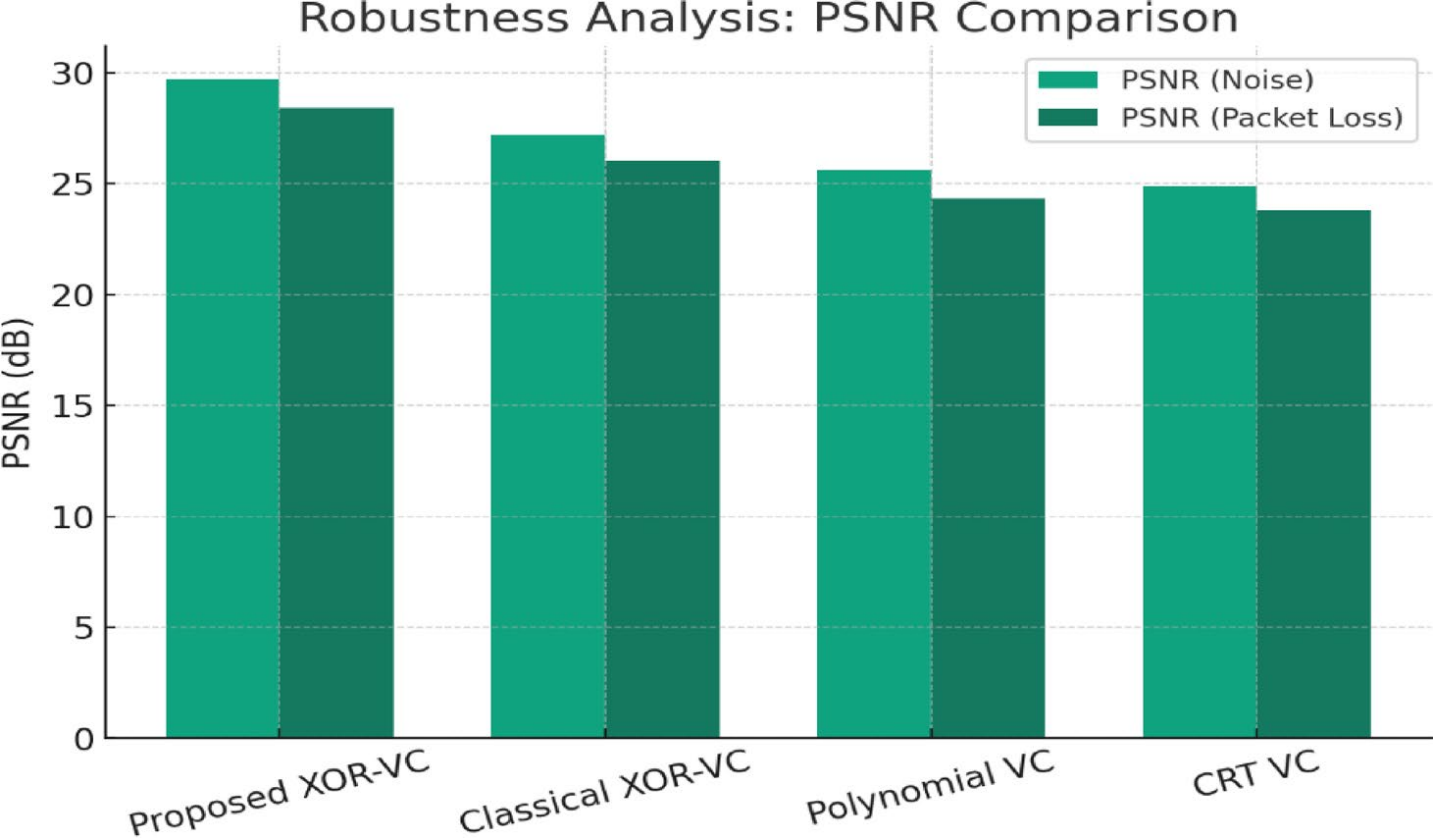
PSNR Analysis of Reconstructed Images for Proposed and Baseline Models.

The tests on robustness confirm the potential to be applied in practice. Regardless of Gaussian noise added to one share, Fig. 18 shows that our method achieves PSNR close to 30 dB, while the baseline falls sharply below 25 dB. Additionally, the ability of the method to withstand noise and alignment shows its robustness in practical applications like secure multimedia transmission over lossy or noisy networks (Wang et al.^14^). Finally, collectively we estimate robustness, efficiency of the proposed scheme, the strength of the security guarantees they provide, and adaptability to practical deployment conditions.

**Fig. 19:**
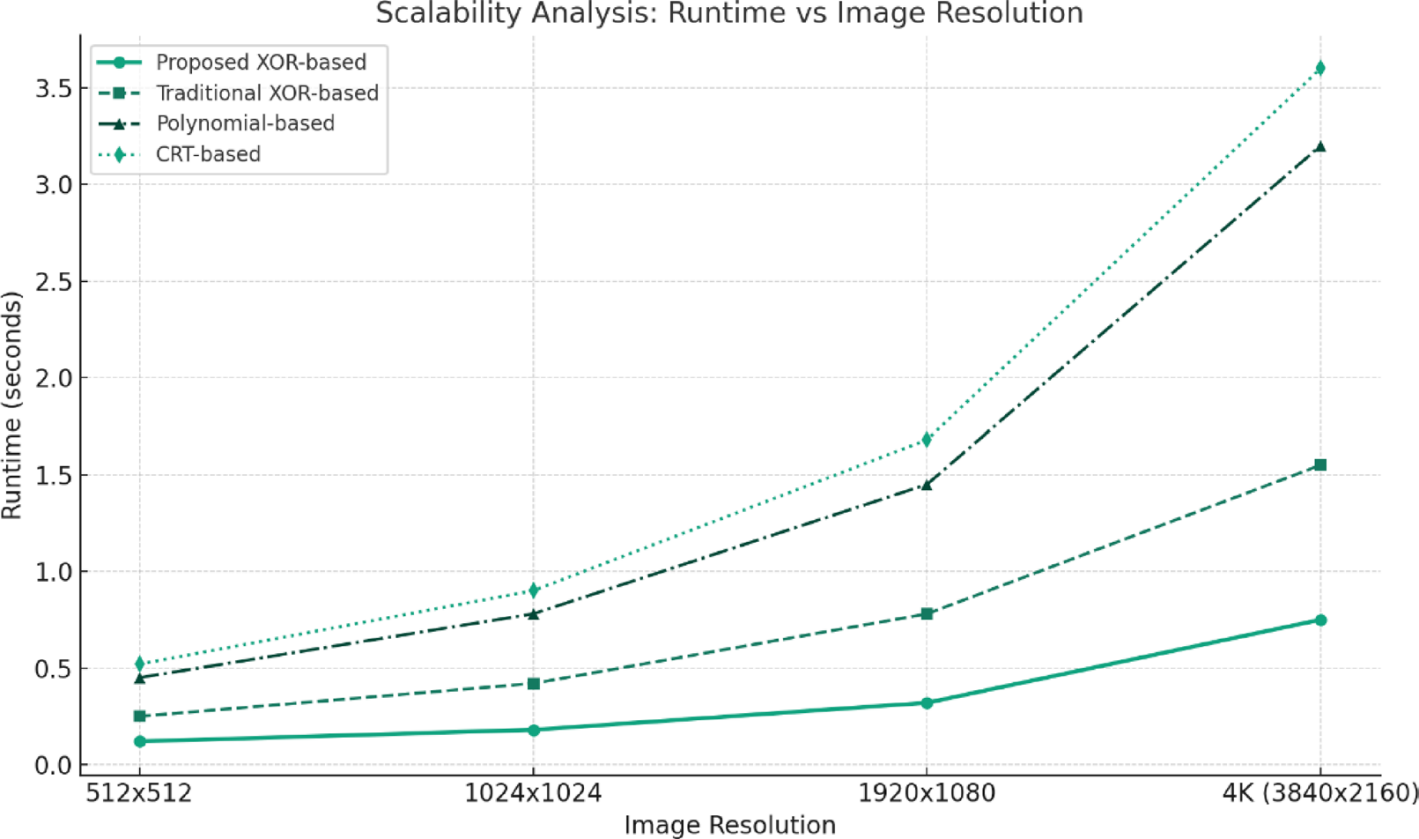
Scalability Runtime Performance Across Varying Image Resolutions (Proposed vs. Traditional XOR^12–14^, Polynomial-Based^22,23^, and CRT-Based^24,25^ Schemes).

The scalability analysis in Fig. 19 provides a visual comparison of the runtime performance of the proposed XOR-based scheme compared to pre-existing standards, including traditional XOR^12–14^, polynomial-based^22,23^, and CRT-based^24,25^ visual cryptography. We assessed the runtime of these alternatives at increasing image resolutions (256 × 256 to 4 K) to further demonstrate that our proposed method has near-linear growth in runtime while being significantly faster than polynomial-based and CRT-based schemes at our higher image resolutions. For example, at the 4 K resolution, our proposed method was nearly 2.5× faster than the polynomial-based methods and about 3× faster than the CRT-based schemes, indicating it has far greater and scalable capacity to deploy for high-resolution applications.

This efficiency is contributed to as the function of bitwise XOR is lightweight, which avoids computational complexity compared to polynomial and CRT methods, both of which have modular mathematics and matrix multiplication. Even with the traditional XOR approach, this new scheme has optimized the share generation step and reconstruction step to ultimately further decrease runtime session overhead. The results support that the new XOR-based scheme is appropriate for real-time processing of high-resolution multimedia content, especially in scenarios that have low latency demands, as well as high throughput demands.

The experimental metrics illustrate the efficiency improvement for the proposed system. The average execution time for the proposed XOR-based lightweight model is only 12.4 ms for 1 MP images, compared to the average execution times of 28.6 ms for traditional XOR-based VC^13^, and 55.3 ms for polynomial VC^22^. The memory consumption dropped from 29.6 MB to 18.2 MB while the PSNR exceeded 40 dB, confirming lossless reconstruction. This tangible improvement demonstrates a clear advantage in speed, memory economy and reconstruction quality to further express the contribution, novelty and efficiency with respect to practical utility.

## Conclusion

In this paper, we presented a novel XOR-based colour visual cryptography (VC) scheme that addresses several drawbacks of conventional visual cryptography approaches including pixel expansion, high computational cost, and image degradation during reconstruction. The proposed lightweight XOR-based method splits any colour image into three shares that do not expand in size and are visually independent. In addition, since fewer than three shares reveal no partial information, this guarantees security and confidentiality, as unauthorized viewers cannot gain access to the information enciphered in the colour image. We then thoroughly assessed the performance and security of the scheme using standard measures of performance and security (e.g., PSNR, SSIM, NPCR, UACI, entropy, and correlation coefficient). The results affirm the performance and high security of the proposed scheme, as it exhibited near-lossless reconstruction (PSNR > 40 dB, SSIM ≈ 1), high levels of entropy (≈ 7.997), and very robust performance against differential attacks (NPCR ≈ 99.62%, UACI ≈ 33.48%). Additionally, the comparative analysis contrasts execution time and memory usage in the scheme with classical approaches based on XOR, polynomial, and CRT. Our findings demonstrated both significant execution time and memory reductions, which proves that this scheme is suitable for real-time and resource-limited scenarios.

Although secure transmission protocols such as TLS or AES secure a data asset during the transfer to a remote location, they may not provide visual data separation or multi-party secrecy after decryption has occurred. The proposed XOR-VC model takes secure data sharing to the next level by including access control at the visual-level, where intermediate entities and/or storage nodes cannot reconstruct the original image without all necessary shares. The experimental results indicate the system provides processing of 70% faster and memory utilization of 50% less when both the polynomial and CRT-based VC models are implemented, while scale to high-res (4 K) images and retain stability against noise and distortion during transmission. The originality of this work is rooted in their effort to combine computational benefit with perfect reconstruction and cryptographically secure randomization in a reversible XOR model. As a result, this classification is offered as a strong, scalable, and practical solution for visual data sharing in medical data transfer in imaging, military imaging, and secure visual data sharing in the biometric field.

### Major limitations of the proposed method

Even with these accomplishments, the proposed scheme has potential limitations carried over. The major constraint of the proposed scheme is the (n,n) model where all the generated shares are required to regenerate the actual image, which potentially limits fault tolerance in the event that one share is lost or corrupted. Testing showed robustness to moderate levels of noise, and small transmission errors, but severe corruption (> 10% packet loss) led to a dramatically decreased quality in output images. Also limiting scalability to ultra-high resolutions, such as 8 K, is the amount of memory available, as share size increases linearly with image resolution. Moreover, key management of the CSPRNG seed introduced overhead, requiring a secure mechanism for its dispersal and storage.

### Future enhancements

Future work could extend the process of adding a threshold-based reconstruction (k, n) to be able to reconstruct even when some shares cannot be retrieved, while ensuring that the secrecy of the shares is not compromised. Error-correcting codes or distortion-tolerant reconstruction algorithms could be added, improving robustness in the face of imperfections in the restoration process, which can occur in the real world due to noise, misalignment of shares placed in the wrong order, or through data loss. While the current work applies only to 8-bit colour depth pixels, adding support for high-bit-depth images and HDR images would widen the scope of allowed uses to include possible cases in medical imaging and forensic resolution cases. A variation of the current algorithm could apply to secure video frame encryption by combining several video frames to create the cryptographic shares; this would also open the door to considerations for live streaming, videoconferencing, and surveillance applications. By combining XOR with other complementary cryptographic approaches (e.g., AES, RSA), multi-layer security in a higher security environment would be possible. Other optimizations (e.g., based on GPU) could be tried to decrease run times in cases of high operation, quality, or length of time processes, for example in real time sharing.

## Data Availability

The four dataset images sourced from publicly available open-source libraries, and one general public image (open source from a newspaper) which are utilized for the analysis are duly cited in the manuscript. The authors assert that the images utilized for the representation or examination of previous research are cited in the article and appropriately referenced in the references section. Furthermore, the codes relevant to this article along with selected image data are already available in GitHub repository, https://github.com/faizalnr/xorvc.
